# Emergent Headwater Types for Watershed Monitoring, Research, and Planning

**DOI:** 10.1029/2026GL123244

**Published:** 2026-09-10

**Authors:** E. C. Seybold, J. Hammond, A. Husic, H. McMillan, H. E. Golden, J. R. Christensen, C. Segura, M. Zimmer, A. N. Price, K. L. Jaeger, L. Li, D. T. Mahoney, F. Cheng, C. N. Jones, C. Kelleher, R. Sando, C. R. Lane, A. Ward

**Affiliations:** 1 Kansas Geological Survey, University of Kansas, Lawrence, KS, USA,; 2 Department of Geology, University of Kansas, Lawrence, KS, USA,; 3 U.S. Geological Survey, Maryland-Delaware-D.C. Water Science Center, Baltimore, MD, USA,; 4 Department of Civil and Environmental Engineering, Virginia Tech, Blacksburg, VA, USA,; 5 Department of Geography, San Diego State University, San Diego, CA, USA,; 6 U.S. Environmental Protection Agency, Office of Applied Science and Environmental Solutions, Cincinnati, OH, USA,; 7 U.S. Environmental Protection Agency, Office of Wetlands, Oceans and Watersheds, Cincinnati, OH, USA,; 8 Department of Forest Engineering, Resources, and Management, Oregon State University, Corvallis, OR, USA,; 9 Soil and Environmental Sciences Department, University of Wisconsin, Madison, WI, USA,; 10 USDA Forest Service Pacific Northwest Research Station, Portland, OR, USA,; 11 U.S. Geological Survey, Washington Water Science Center, Tacoma, WA, USA,; 12 Department of Civil and Environmental Engineering, The Pennsylvania State University, State College, PA, USA,; 13 Department of Civil and Environmental Engineering, University of Louisville, Louisville, KY, USA,; 14 Department of Environmental Sciences, University of Virginia, Charlottesville, VA, USA,; 15 Biological Sciences, University of Alabama, Tuscaloosa, AL, USA,; 16 Department of Civil and Environmental Engineering, Lafayette College, Easton, PA, USA,; 17 U.S. Geological Survey, Wyoming-Montana Water Science Center, Helena, MT, USA,; 18 Lane Ecological Advisory LLC, Pewaukee, WI, USA,; 19 Biological and Ecological Engineering Department, Oregon State University, Corvallis, OR, USA

## Abstract

Headwaters—that is headwater streams and the watersheds they drain—strongly influence water quality, ecosystem services, and hydrologic connectivity, yet remain poorly mapped, monitored, and understood at large spatial scales. Using available spatial data to classify headwater “types” could therefore enhance the utility of sparse monitoring data to advance research and watershed planning—and help update literature-based perceptual models of headwaters as steep, forested, and minimally disturbed watersheds. Here, we analyze headwaters across the contiguous United States to quantify how their attributes (climate, topography, land cover, soils) vary regionally and differ from their downstream watersheds. Our analysis (a) identified seven dominant headwater types with distinct combinations of biophysical and climatic attributes and (b) revealed systematic contrasts with downstream watersheds, with strongest differences in attributes representing potential flowpath depth. Our findings offer a transferable framework for classifying, modeling, and managing these critical yet understudied components of the hydrologic network.

## Plain Language Summary

Headwater streams are small, represent the “starting point” of watersheds, and exist in the uppermost portions of a stream network. Their size, prevalence, and location in watersheds make them essential for clean water supplies and ecosystem health. However, headwater streams and the watersheds they drain are rarely mapped or studied across large geographic extents, leaving major gaps in our understanding of how they function across the United States. In this study, we examined how physical characteristics like climate, topography, land cover, and soils vary among headwaters across the contiguous United States (CONUS) and generated a classification system of headwater “Types” using these characteristics. We further compared these headwater characteristics to those of their downstream watersheds. We found clear and consistent patterns in physical characteristics regionally among headwater types and between headwaters and their downstream watersheds. These results provide a national-scale snapshot of headwater diversity and offer new tools to guide watershed management and conservation in changing environments.

## 1. Introduction

Headwater streams and headwater systems provide core watershed functions, including water quality maintenance, flood and drought attenuation, and ecosystem services (Finn et al., 2011). Headwater streams are the upstream-most visible channels with flowing waters for at least part of the year, and headwater systems, (i.e., headwater watersheds) are “catchment areas and groundwater zones contributing water, [solutes, sediment, wood], and energy to a headwater stream” (Golden et al., 2025; Wohl et al., 2015). Collectively, we term these areas headwaters.

Headwaters are dynamic, with highly variable flow regimes, yet vulnerable to degradation (Lane et al., 2023) because they are sparsely mapped, measured, monitored, and modeled. This lack of information limits protective actions worldwide (Kampf et al., 2021; Wohl, 2017), and improved scientific understanding of these systems is essential to support management. Headwaters comprise ~77% of CONUS stream lengths (Lane et al., 2026)—making them too abundant and widespread to monitor and manage individually. Researchers and watershed managers therefore require ways to (a) regionally characterize headwaters based on their physical characteristics and (b) understand what distinguishes them from their downstream waters.

Our understanding of headwater hydrology, biogeochemistry, and ecology derives from decades of research primarily in long-term, well-monitored forested watersheds (Gomi et al., 2002). The United States (US) Forest Service began developing experimental forests in the early 20th century (Bogena et al., 2018; Campbell et al., 2021; Miniat et al., 2021) to evaluate forest management and water yield from small headwaters in forests and rangelands (Sebestyen et al., 2025). Thus, our perceptual models of headwater hydrologic processes—such as runoff generation (Burt & McDonnell, 2015), evapotranspiration (Bosch & Hewlett, 1982), and subsurface flow (Ferguson and Maxwell, 2010; McGlynn et al., 2002)—are largely (61%; based on McMillan et al., 2025) based on forested catchments (Table S1 in Supporting Information S1). More recent headwater research has expanded beyond steep, forested, minimally impacted systems into agricultural and urban systems, such as the Long-Term Agricultural Research network (LTAR; Goodrich et al., 2021) and new inclusions of agricultural and urban watersheds in the Long-term Ecological Research (LTER, Grimm et al., 2000; Pickett and Cadenasso, 2006) and Critical Zone Observatory (CZO) networks (Singha et al., 2024). However, these heavily impacted headwaters remain underrepresented in monitoring and process understanding (Bieroza et al., 2024; Imberger et al., 2023; Singha et al., 2024).

Headwater hydrological and ecological processes can vary widely (Becker, 2005; Buttle et al., 2012; Jensen et al., 2017; McManamay and DeRolph, 2019; Penna, 2026). Yet we lack synthetic, continental-scale analyses demonstrating how their characteristics vary and how they differ from their downstream watersheds—important information to broaden and update our perceptual models and to support future headwaters research and watershed planning. New approaches to headwater characterization that rely on readily available, large-scale data sets, rather than on limited streamflow records alone (Hasan et al., 2024), are needed to understand headwater stream structure and function (Ciulla and Varadharajan, 2024; Cox et al., 2023). Regional-scale schema, sensu hydrologic landscape regions (Winter, 2001) demonstrate the value of such watershed classification approaches for scientific and management applications (Ciulla and Varadharajan, 2024; Wagener et al., 2007; Wolock, 2003).

Here, we develop a national synthesis of headwaters to provide an operational framework for transferring headwater process knowledge, management strategies, and modeling approaches to diverse regions of the contiguous United States (CONUS). In this paper, we address these scientific and management needs by answering the following questions:

1. How do headwater attributes vary across the CONUS?
2. How do headwater attributes compare to their downstream watersheds?

## 2. Data and Methods

We leveraged a new CONUS-wide data set that maps the extent of headwater streams (Lane et al., 2026) to extract headwater attributes. This high-resolution data set builds upon the U.S. National Hydrography Data set Plus High Resolution (NHDPlus-HR; 1:24000; USGS, 2019), resolving previous topological irregularities and providing a spatially consistent delineation of headwaters across diverse physiography (Lane et al., 2026). Because headwater definitions vary, we adopted a consistent operational definition (Golden et al., 2025; Lane et al., 2026). We define headwater watersheds as those areas draining NHD Strahler stream orders 1 and 2 (SO1&2, from here on termed “headwaters”), and downstream watersheds as those draining orders 3 and greater but excluding the upstream headwaters (SO ≥ 3, from here on termed “downstream”) (Figure S1 in Supporting Information S1). We discretized the CONUS into 12-digit hydrologic unit code (HUC12) areas—~82,000 HUCs—each containing multiple headwater and downstream areas.

Our workflow included four steps: (a) calculate spatially averaged attributes for headwaters and downstream in each HUC12, (b) classify headwaters using clustering analysis, (c) explore emergent characteristics of each headwater type, and (d) assess how attributes differ between headwaters and downstream using random forest models.

### 2.1. HUC12 Watershed Attributes

We identified 82,213 HUC12 watersheds with boundaries entirely within the CONUS and that included complete SO1&2 NHD flowlines. HUC12 watersheds ranged from 0.001 to 7,133 km^2^ (mean 63.9 km^2^). For each HUC12, we delineated headwaters and downstream areas (Figure S1 in Supporting Information S1) and summarized climatic, topographic, soils, land cover, and hydrologic attributes (Table S2 in Supporting Information S1) as the mean across the respective headwater or downstream watersheds using zonal statistics in R (v4.2.2; R Core Team, 2022) via the exactextractr package (Baston, 2023).

We used attributes similar to those in GAGES-II (Falcone, 2011), given its wide application in hydrologic analyses (Addor et al., 2018; Hammond et al., 2021; Hodgkins et al., 2019; Konapala and Mishra, 2020). We selected variables that (a) represent hydrologically relevant processes, (b) were not highly correlated (|r| < 0.8), (c) are publicly available, and (d) do not rely on direct hydrologic measurements. Attribute selection followed best-practice guidelines emphasizing process representation, spatial heterogeneity, and uncertainty considerations (Tarasova et al., 2024). The 16 selected attributes (Table S2 in Supporting Information S1) represent climate (aridity index, snow persistence index); topography (watershed slope and elevation); soils and geology (% sand, % clay, depth to bedrock); land cover and human-related alteration (% forest, % developed, % water/wetland, % agricultural, % shrub/grass, population density); and hydrology (% surface, shallow, and deep Height Above Nearest Drainage [HAND] class).

A key aim was to include attributes that characterize hydrological function of headwaters, including partitioning, storing, and release of water (Black, 1997; Wagener et al., 2007). These functions emerge, in part, from water flowpaths: surface, shallow subsurface, or deep groundwater. The HAND metric quantifies the topographic relief along the drainage network, and therefore the relative soil gravitational potentials or local draining potentials (Nobre et al., 2011). HAND correlates strongly with water table depth and has been used to infer runoff generation mechanisms (Savenije, 2010). We therefore used HAND as a proxy for potential flowpath depth and, when combined with information about soil type and bedrock depth, to provide information on water partitioning.

Each headwater watershed was discretized into three HAND classes. Low HAND values (<5 m, surface flowpaths) and clay-rich soils suggest areas of saturation overland flow in valley bottoms (e.g., Peterson et al., 2025). Mid HAND values (5–15 m, shallow flowpaths) represent areas of subsurface stormflow along hillslopes (e.g., Zimmer and McGlynn, 2017). High HAND values (>15 m, deep flowpaths), sandy soils, and deep bedrock reflect areas of deep infiltration and groundwater flow on ridges or plateaus (e.g., Selker, 2017). However, as a topographic metric, HAND omits subsurface features that influence specific flow conditions like stream drying (e.g., Glaser et al., 2025). We refer to HAND variables as percent surface, shallow, and deep flowpaths; however, they are ultimately a proxy for *potential* flowpath depth.

### 2.2. Clustering Methods

We used watershed attributes to classify headwaters across the CONUS and develop a framework for transferring physical headwater characteristics to future modeling applications. The goal was to identify a relatively small number of headwater types that capture key physiographic and climatic gradients while remaining interpretable for large-scale modeling. The clustering approach classifies CONUS headwater landscapes, similar to other frameworks such as U.S. Environmental Protection Agency Level I Ecosystems (Omernik and Griffith, 2014) and Hydrologic Landscape Regions (Wolock et al., 2004).

We used k-means clustering (scikit-learn; Pedregosa et al., 2011) on normalized and transformed attributes to identify types of headwaters with similar climatic, subsurface, and land-cover characteristics. K-means clustering is an unsupervised clustering algorithm that iteratively assigns observations to the nearest of *k* cluster centroids by minimizing within-cluster variance and recalculates centroids until convergence (MacQueen, 1967). Prior to clustering, the Yeo–Johnson transformation (Yeo & Johnson, 2000) was applied to correct skewness and standardize variables (zero mean, unit variance).

To identify the optimal number of clusters (*k* = 1–15), we used elbow plots of the within-cluster sum of squares and silhouette scores to compare within- and between-cluster variance (Text S1, Figures S2 and S3 in Supporting Information S1). Based on these assessments, we selected 7 clusters to balance parsimony and interpretability. Tests showed that 6–8 clusters produced similar spatial distributions (Figure S4 in Supporting Information S1); using 8 clusters simply created smaller regional sub-groups. As in Wolock et al. (2004), our final classification combines data-driven clustering with expert interpretation to minimize within-cluster variance while ensuring applicability at the CONUS scale.

### 2.3. Comparison of Watershed Attributes Across Clusters and Scales

We visualized differences among headwater clusters, which will henceforth be referred to as headwater “types,” by computing mean z-score values of each attribute (7 types × 16 attributes = 112 combinations) and mapping their spatial distribution. To evaluate contrasts between headwaters and downstream, we used the Kolmogorov-Smirnov test (Massey, 1951; *α* = 0.5) to compare cumulative attribute distributions for each headwater type.

### 2.4. Random Forest Analyses

To quantify how headwaters differ from downstream, we trained a random forest model to classify watersheds as (a) headwater (SO1&2) or (b) downstream (SO ≥ 3) using the 16 watershed attributes as predictors. The dependent variable was the categorical label (headwaters or downstream). Significant differences in the predictors helped explain contrasts between headwaters and downstream. One global model was trained across all watersheds using bootstrapped samples to reduce overfitting, and predictions were determined by majority vote among trees.

Model performance was validated using out-of-bag error estimates, and we optimized model complexity (number of trees and maximum node splits) to minimize classification error and maximize the mean margin (Breiman, 2001; Husic et al., 2025; Figures S5 and S6 in Supporting Information S1). A receiver operating characteristic (ROC) curve quantified trade-offs between true and false positive rates (Fawcett, 2006; Figure S7 in Supporting Information S1). All random forest analyses used the fitcensemble function in MATLAB version: R (2024).

We used Shapley values (ψ) (Husic et al., 2025; Lundberg & Lee, 2017) to interpret variable influence on model predictions. Global attribute importance was calculated as the mean absolute Shapley value (|ψ|), and we evaluated variation across the seven headwater types to identify type-specific drivers. Shapley dependence plots illustrated how key attributes affected model predictions, and spatial maps of Shapley values revealed geographic variation in attribute effects. All Shapley analyses used the shapley function and TreeSHAP algorithm in MATLAB version: R (2024).

## 3. Results

### 3.1. Variations in Headwater Characteristics Across the CONUS

We identified seven distinct headwater types across the CONUS (Figure 1) with varying watershed attributes (Figure 2; Figures S8–S10 in Supporting Information S1). Types 2 and 7 most closely match historical perceptual models of forested headwaters (McMillan et al., 2025): both are forested, steep, and have a high topographic potential for deep flowpaths and deeper groundwater tables. Types 2 and 7 differ mainly in climate, subsurface traits, and anthropogenic impact. Type 2 has low snow persistence, higher clay percent, and greater agricultural land cover and population density; type 7 has high snow persistence, higher sand fraction, and lower agricultural land cover and population density.

Types 1 and 3 are also forested, but have lower slopes with high wetland/open water coverage. They have high percent sand, low slope and elevation, shallower bedrock depths, and higher percent of potential surface flowpaths. This suggests that shallow water table dynamics could be driven by topography and bedrock depth, but offset by sandy soils. These types differ largely by their climate: Type 1 has high snow persistence, while Type 3 has low snow persistence.

Types 4 and 5 are most defined by land cover and subsurface characteristics and span a wide climatic range (e.g., snow persistence index). Type 4 has more agriculture with clayey soils, deep bedrock, and a high percentage of potential surface flowpaths, suggesting that topography and soil type may outweigh bedrock depth in driving surface flowpath dynamics. Type 5 is dominated by shrub/grassland, deep bedrock, and variable HAND classes. Type 6 is the most arid, characterized by shrub/grassland, sandy soils, deep bedrock, and high percent deep potential flowpaths.

### 3.2. Variation in Watershed Attributes Between Headwaters and Downstream

We found significant differences across most attributes (93% of 112 comparisons; Figure 3) between headwaters and downstream, especially for attributes representing hydrologic processes. Across all types, headwaters had greater potential for deep flowpaths, with more high HAND areas and fewer low HAND areas. Differences in potential shallow flowpaths varied: Types 3 and 4 had higher values in headwaters, while other types had higher values downstream.

Differences in land cover attributes between headwaters and downstream were strong, especially for the percentage of open water/wetland class, developed land, and forest cover. However the direction of differences in land cover attributes was mixed; water percent is lower in all headwater types, while forest and shrub/grass cover are higher in headwaters for most types. Topographic and climatic differences were weaker than hydrologic and land cover differences, but were notable for bedrock depth, elevation, slope, and snow persistence. Headwater elevations and slopes were higher across nearly all types.

### 3.3. Random Forest Classification Model to Predict Headwater vs. Downstream Watershed

The random forest model accurately distinguished headwaters from downstream (out-of-bag accuracy = 0.84; misclassification fraction = 0.158; AUC = 0.91; Figures S5–S7 in Supporting Information S1). Shapley values (ψ) identified percent surface and deep flowpaths, mean slope, and percent water cover as the most important predictors (Figure 4). We sub-sampled Shapley results from the overall model to show differences in attribute importance by headwater type (colored circles in Figure 4). Higher values than the overall model suggest that the attribute was more influential in determining classification (headwater or downstream) for that headwater type. For example, the percent surface flowpath attribute was more influential for Types 2 and 7 than the overall model, and less influential for Types 1, 4, 3, and 5.

Shapley dependence plots reveal how key attributes influence classification across their range (Figure S11 in Supporting Information S1). Low potential for surface flowpaths and low (<2%) open water fraction increase the probability of headwater classification. Land cover of more than 2% water increases the probability of downstream classification. Interestingly, the model interprets low mean watershed slope as increasing the probability of headwater classification, particularly below 2°. This is partly because, for locations with low overall slopes such as the Midwestern USA (Figure S12 in Supporting Information S1), the slope of downstream watersheds is actually slightly greater than that of headwaters. In steeper locations, other features such as percent potential surface flowpaths contribute to the prediction of a basin as a headwater more than slope (Figures S11 and S13 in Supporting Information S1).

## 4. Discussion

### 4.1. Variability in Headwater Types

Headwaters across the CONUS are highly diverse. Emergent headwater types vary spatially (Figure 1) and differ in potential hydrologic flowpaths, topographic slope, aridity, snow persistence, and land cover (Figure 2). These seven types emphasize that most CONUS headwaters differ from the steep, forested watersheds frequently found in historical literature (e.g., Beven et al., 1988; Kirchner, 2009; Pilgrim et al., 1982; Refsgaard et al., 2022). Perceptual models stemming from long-term research in such watersheds (e.g., HJ Andrews (Oregon), Coweeta (North Carolina), Hubbard Brook (New Hampshire)) may not represent hydrological processes occurring in ~70% of headwater clusters not represented by these conditions.

Our findings, therefore, highlight the need to expand beyond monitoring in—and dominant perceptual models of—headwaters in steep, forested, low-impact systems (McMillan et al., 2025). Only two headwater types (2 and 7, which comprise about ~30% of CONUS headwaters) represent this dominant model. Yet, ~66% of streamflow data are collected in headwater Types 2 and 7 (based on Sando et al. (2026; Figure S14 in Supporting Information S1), signaling that other headwater types are underrepresented in current data. Other types—many forested (e.g., 1 and 3)—have lower slopes, greater surface water storage, and higher potential for shallow flowpaths, including some experimental forests like Sleepers River (Shanley et al., 2022), Marcell (Sebestyen et al., 2021), and Panola Mountain (Aulenbach et al., 2021)). Together, data from long-term study sites outside forested systems (e.g., Type 4—Intensively Managed Landscapes CZO, Type 5—Konza Prairie LTER, Type 6—Sevilleta LTER) can help build a comprehensive understanding of hydrologic processes across headwater types. In short, our headwaters classification system can support strategic selection of long-term monitoring sites where data are sparse to assist in understanding headwater functions (Golden et al., 2025).

Most headwaters are not minimally impacted. No single headwater type was dominated by developed (urban/suburban) land cover; all include some (median of 8%–12% in Types 1–4, Figure S9 in Supporting Information S1). Given the high percent of agricultural land cover across headwater Types 1–5 (Figure S9 in Supporting Information S1), our findings align with emerging work showing that agricultural headwaters are often overlooked (Bieroza et al., 2024). Therefore, inclusion of anthropogenic processes could improve the accuracies of models describing headwater streamflow dynamics.

Clear regional patterns and deviations emerged. Type 7 headwaters (steep, snowy, deep potential flowpaths) dominate the western U.S. (e.g., Rocky Mountains), but also occur in the eastern U.S. (e.g., Appalachian Mountains and White Mountains). Type 2 (steep, forested, temperate) headwaters are common in the eastern U.S. and appear in unglaciated regions of the Upper Midwest and Pacific Northwest. This framework identifies where headwaters deviate from regional expectations (e.g., Type 7, dominant in mountains of the western U.S., is scattered in small portions of the Eastern U.S.) and shows that analogous headwaters can be geographically diverse.

### 4.2. Potential Flowpath Depths Distinguish Headwaters From Downstream Drainage Areas

Headwaters and downstream watersheds have systematically different distributions of watershed attributes, as revealed by high identification accuracy (Figure S5 in Supporting Information S1). Potential flowpath depths (inferred by dominant HAND classes) have the strongest predictive power between headwaters and downstream (Figures 3 and 4), demonstrating that differences in the topographic potential for varying flowpath depths overrides differences in climate and land cover. Headwaters have lower potential for surface flowpaths and greater potential for deep flowpaths, which may arise from steeper valleys and narrower floodplains (e.g., in Types 2 and 7). However, potential flowpaths for Types 1 and 4 are less differentiated from their downstream watersheds (lower magnitude KS d-statistic). These types are humid with low slopes and higher potential for surface flowpaths in the headwaters. While HAND metrics provide interpretable patterns, they are limited by resolution and uncertainties in the underlying elevation and NHD hydrography data sets, including lack of headwater stream representation that could bias HAND estimates toward deeper flowpath categories. Despite potential limitations, HAND provides a comprehensive proxy for potential hydrologic dynamics that can be validated at the watershed scale. Incorporation of distinct runoff generation pathways in headwaters versus downstream into modeling tools is essential for accurate predictions of streamflow and water provisioning (Kim et al., 2022).

### 4.3. Implications for Headwater Monitoring, Research, and Planning

Our findings underscore that data from historical experimental watersheds may be insufficient to inform modeling, monitoring, and future research and watershed planning in diverse CONUS headwaters. Robust monitoring of headwaters would require substantial resources, time, and effort that, in practice, are limited. Therefore, frameworks that group headwater types based on their dominant features and identify regional differences from their associated downstream systems may help guide future monitoring. Moreover, variations in topographic and climatic attributes across headwater types—and between headwaters and downstream—challenge the idea that management decisions made at regional or large watershed scale sufficiently address headwaters. For example, multiple headwater types present across the Pacific Northwest (primarily in the Columbia River Basin) are managed on a state-by-state basis without explicit consideration of headwater functions and attributes (Kampf et al., 2021).

Models struggle in headwaters (Golden et al., 2025), an outcome that is compounded by lack of data (Beven, 2024; Golden et al., 2025; Pandit et al., 2025; Sando et al., 2026). Because headwater types vary widely, there is likely no “best” hydrological model for headwaters. Instead, our results may help identify important hydrological processes across headwater types to inform future data collection and model selection.

### 4.4. Further Analyses Are Needed to Link Headwater Types and Hydrologic Function

Future research is needed to quantitatively assess how differences in watershed attributes across headwater types translate into variable hydrologic function. For example, the seven headwater types differ substantially in the percent of stream lengths classified as non-perennial and perennial in the NHD (Figure S15 in Supporting Information S1). Streams in Type 6 watersheds are almost entirely non-perennial, while Type 1 watersheds are almost entirely perennial. This suggests that distinct watershed attributes describing the seven headwater types translate into distinct streamflow regimes. Future analyses are needed to assess how streamflow regime and predictability vary across headwater types, as streamflow data spanning headwater types become available.

## 5. Conclusions

Our synthesis demonstrates seven headwater types across the CONUS, which differ widely in physiography and characteristics like potential flowpath depth, topography, climate, and land cover. Most headwaters do not resemble the small, steep, forested watersheds frequently found in the historical literature. Only Types 2 and 7 (~30%) represent these conditions, while others are dominated by cultivated crops, grasslands and shrubs in low-gradient terrain (e.g., Types 4, 5, and 7).

HAND class, a proxy for flowpath depth, emerged as a key distinguishing attribute between headwaters and downstream waters, highlighting functional differences that could be incorporated into watershed models. The prevalence of anthropogenic land use across most types emphasizes that human influences are integral to headwater hydrology, modeling, and management.

Our novel headwaters synthesis and unique data set classifying headwaters across the CONUS can therefore guide hydrologic modeling, long-term monitoring, and future research and watershed planning efforts. It can advance our perceptual frameworks to better represent hydrologic structure and function across headwaters. Expanding data collection and model development across underrepresented headwater types will advance our understanding of runoff generation, streamflow regimes, and water quality in these highly diverse regions of the landscape.

## Supplementary Material

Supplement1

1

Supporting Information may be found in the online version of this article.

## Figures and Tables

**Figure 1.**
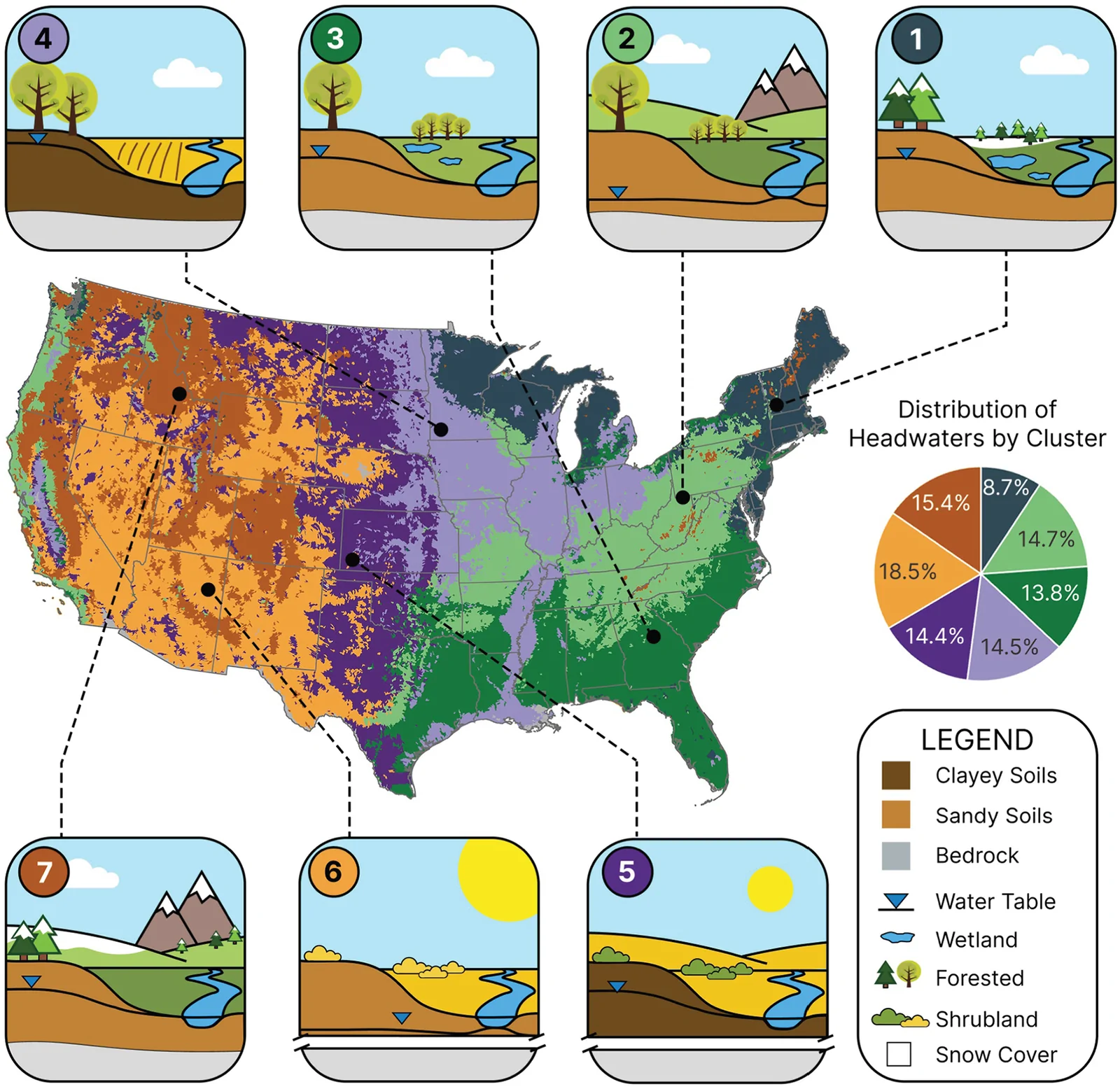
Map of headwater types generated from *k*-means clustering. Each type represents headwater watersheds sharing similar topographic, climatological, subsurface, and hydrological characteristics.

**Figure 2.**
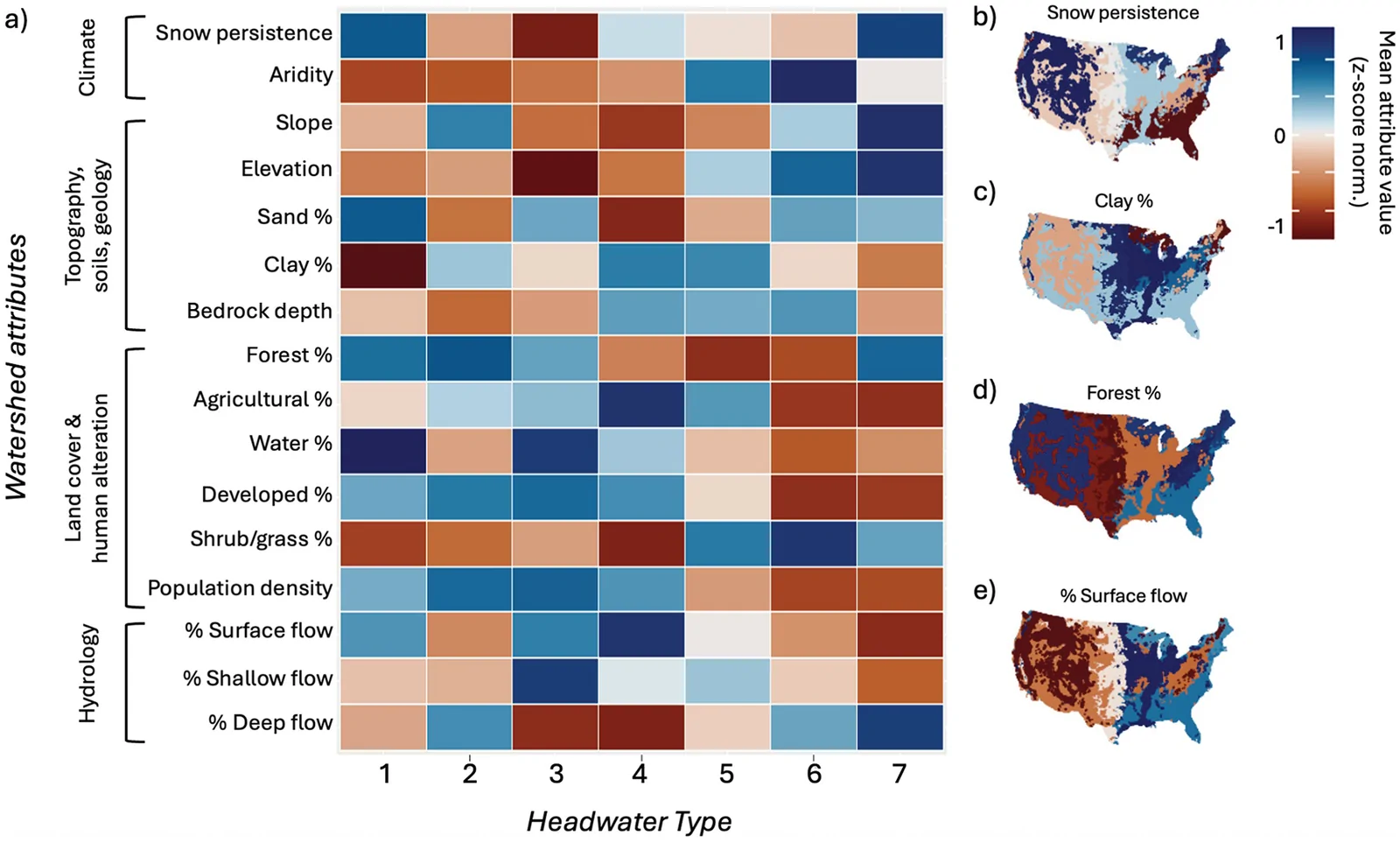
(a) Visualization of mean headwater watershed attributes by cluster. All attribute values have been *z*-score normalized across all clusters. Blue colors indicate that a cluster’s mean attribute value is high relative to other clusters, whereas red colors indicate the cluster’s mean attribute value is low relative to other clusters. Panels (b–e) show the spatial distribution of four selected mean attributes for the 7 headwater types: snow persistence (b), clay % (c), forest % (d), and % surface flow (e). Boxplots displaying data in non-normalized units for all 16 attributes are in Figures S8–S10 of Supporting Information S1.

**Figure 3.**
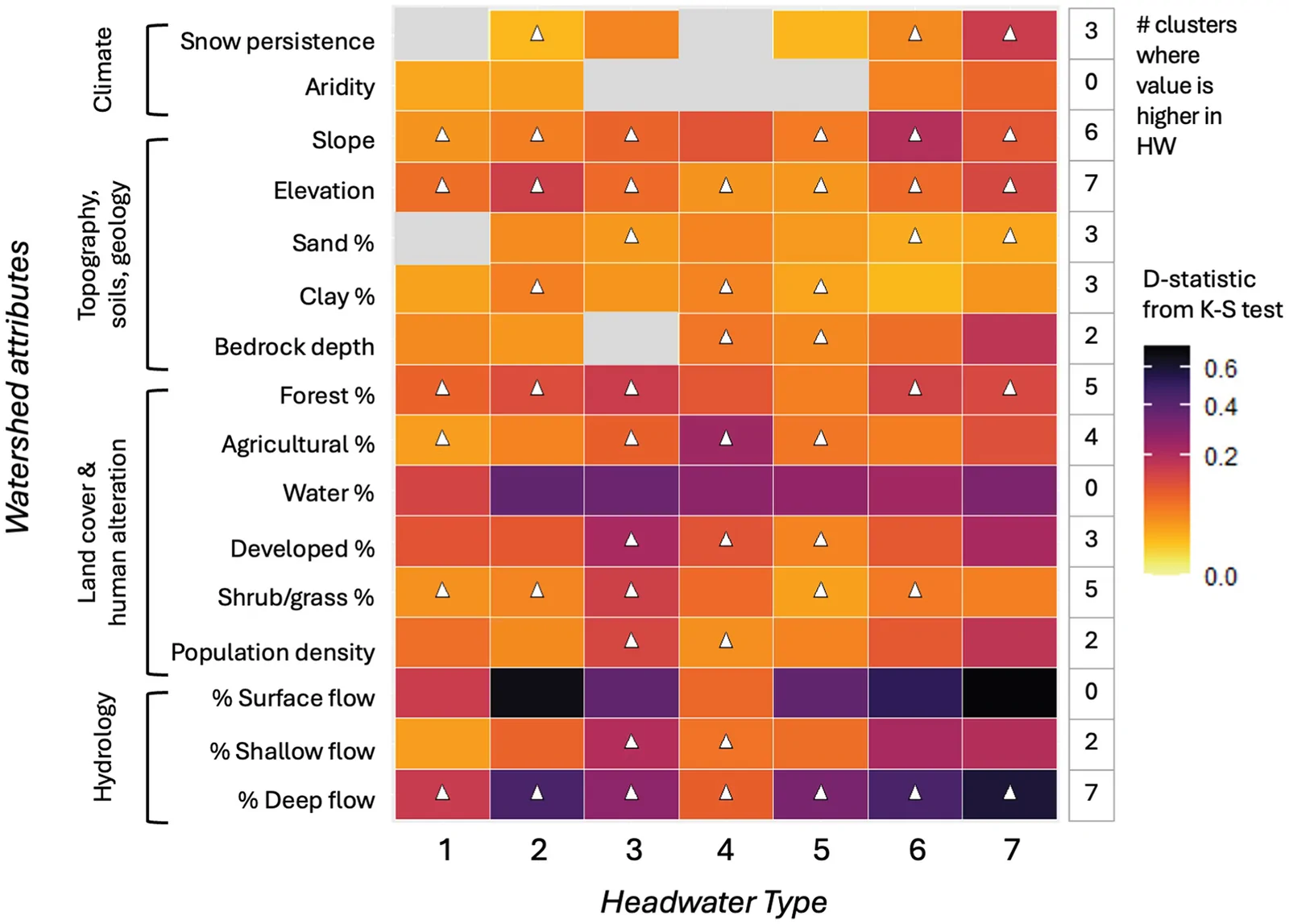
Comparison of watershed attributes between headwater (HW, stream orders 1&2) and downstream (stream orders ≥3) areas for each headwater type (*x*-axis). The color signifies the value of the D-statistic from the K-S test; darker colors indicate higher D-statistic values, indicating greater differences between the headwater and downstream distributions for that attribute. Colored cells indicate significant differences. Upward-oriented triangles indicate clusters where the median attribute value is higher in the headwater than the downstream watershed. Colored cells lacking the triangles indicate that the attribute value is lower in the headwater than the downstream watershed. Cells that are grayed out indicate no statistical difference between headwater and downstream watersheds.

**Figure 4.**
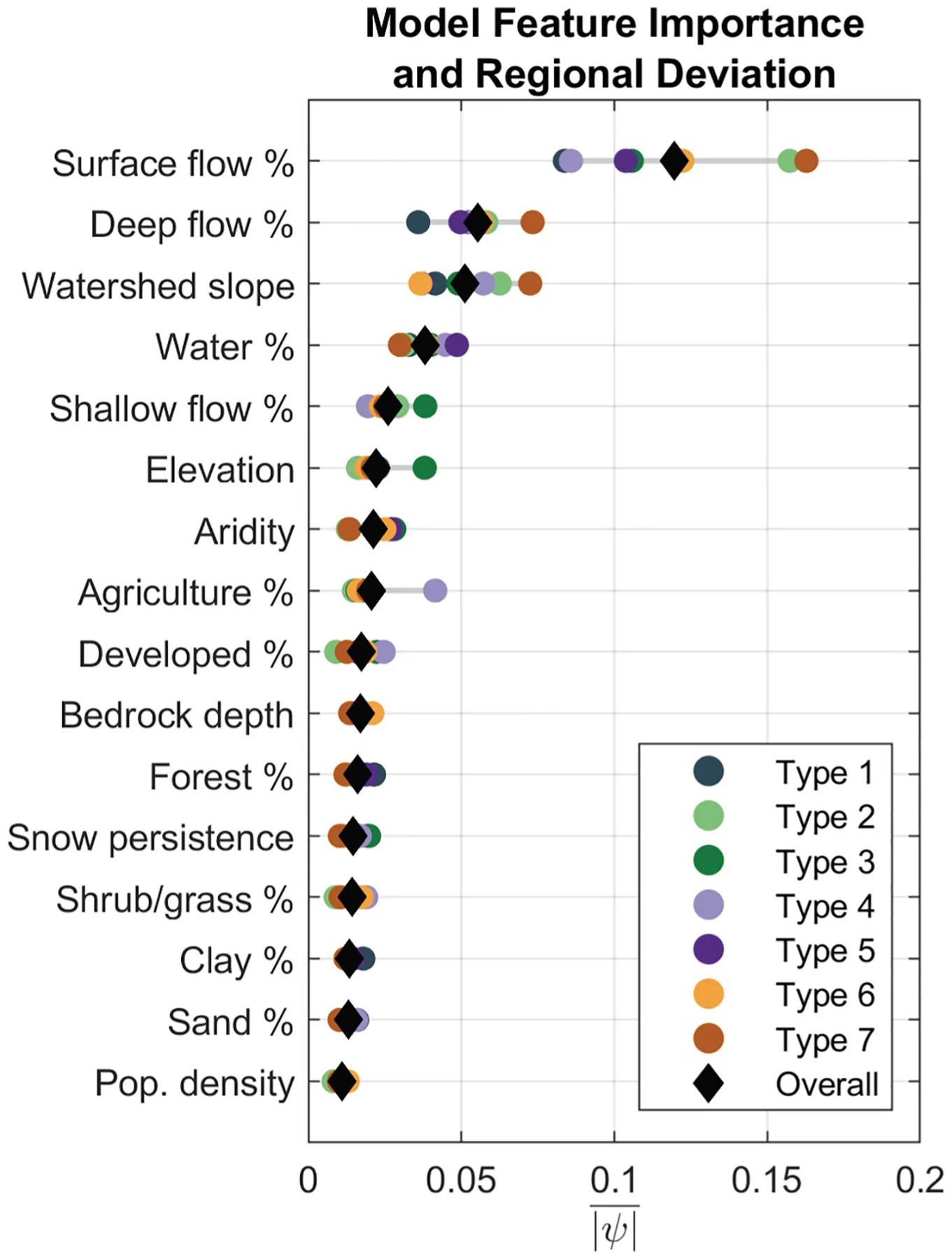
Shapley value (ψ) feature importance for the classification model that classifies watersheds as headwater (stream orders 1&2) or downstream (stream orders ≥3). Feature importance is shown as the mean absolute Shapley value (|ψ|). The Shapley feature importance is noted for the overall assessment (overall, black diamond) and for the importance of the variable within the various clusters (sub-sampled results from the overall mode based on cluster ID).

## Data Availability

All watershed attributes summarized to the HUC12 scale that were used in this analysis are available in Hammond (2025). All data (HUC12 IDs and associated cluster assignments derived from the *k*-means clustering) and code used for spatial extraction of watershed attributes, clustering, and random forest modeling is available in Seybold and Husic (2026).

## Key Points:

- Attributes of headwater watersheds are highly variable across the contiguous United States
- Only 30% of headwaters fit existing perceptual models of forested, steep headwaters, suggesting under-examined variability in many systems
- Headwaters and downstream systems have distinct defining features, with attributes representing potential flowpath depth as key differentiators
