# Supplementary material for: Emergent Headwater Types for Watershed Monitoring, Research, and Planning
Source: Geophys Res Lett. Author manuscript; Available in PMC 2026 Oct 20. (PMC13679705; doi:10.1029/2026GL123244)
Supplement: Supplementary file 1 [file NIHMS2211313-supplement-1.pdf]

10.1029/2026GL123244

19448007, 2026, 17, Downloaded from https://agupubs.onlinelibrary.wiley.com/doi/10.1029/2026GL123244 by USEPA- Office of environmental, Wiley Online Library on [17/09/2026]. See the Terms and Conditions (https://onlinelibrary.wiley.com/terms-and-conditions) on Wiley Online Library for rules of use; OA articles are governed by the applicable Creative Commons License

References From the Supporting Information

Abatzoglou, J. T. (2011). Development of gridded surface meteorological data for ecological applications and modelling. *International Journal of Climatology*, *33*(1), 121–131. https://doi.org/10.1002/joc.3413

Batjes, N. H., Ribeiro, E., & van Oostrum, A. (2020). Standardised soil profile data to support global mapping and modelling (WoSIS snapshot 2019). *Earth System Science Data*, *12*(1), 299–320. https://doi.org/10.5194/essd-12-299-2020

Hammond, J. C. (2020). Contiguous U.S. annual snow persistence and trends from 2001-2020 [Dataset]. *U.S. Geological Survey data release*. https://doi.org/10.5066/P9U7U5FP

Huang, X., Wang, C., Li, Z., & Ning, H. (2021). A 100 m population grid in the CONUS by disaggregating census data with open-source microsoft building footprints. *Big Earth Data*, *5*(1), 112–133. https://doi.org/10.1080/20964471.2020.1776200

Liu, Y. (2023). Height above nearest drainage (HAND) for CONUS [Dataset]. *Hydro*. https://doi.org/10.4211/hs.73aaa3efcda2465ba6227f535400f36b

U.S. Geological Survey. (2024). Annual NLCD Collection 1 Science Products (ver. 1.1, June 2025) [Dataset]. *U.S. Geological Survey data release*. https://doi.org/10.5066/P94UXNTS
