# Supplementary material for: Emergent Headwater Types for Watershed Monitoring, Research, and Planning
Source: Geophys Res Lett. Author manuscript; Available in PMC 2026 Oct 20. (PMC13679705; doi:10.1029/2026GL123244)
Supplement: Supplementary file 2 [file NIHMS2211313-supplement-Supplement1.pdf]

Geophysical Research Letters

Supporting Information for

Emergent headwater types for watershed monitoring, research, and planning

E.C. Seybold[1,2], J. Hammond[3], A. Husic[4], H. McMillan[5], H.E. Golden[6], J.R. Christensen[7], C. Segura[8], M. Zimmer[9], A.N. Price[10], K.L. Jaeger[11], L. Li[12], D.T. Mahoney[13], F. Cheng[14], C.N. Jones[15], C. Kelleher[16], R. Sando[17], C.R. Lane[18], A.S. Ward[19]

[1]Kansas Geological Survey, University of Kansas, Lawrence, KS, USA. [2]Department of Geology, University of Kansas, Lawrence, KS, USA. [3]U.S. Geological Survey, Maryland-Delaware-D.C. Water Science Center, Baltimore, Maryland, USA. [4]Department of Civil and Environmental Engineering, Virginia Tech, Blacksburg, VA, USA. [5]Department of Geography, San Diego State University, San Diego, CA, USA. [6]U.S. Environmental Protection Agency, Office of Applied Science and Environmental Solutions, Cincinnati, OH, USA. [7]U.S. Environmental Protection Agency, Office of Wetlands, Oceans and Watersheds, Cincinnati, OH, USA. [8]Department of Forest Engineering, Resources, and Management, Oregon State University, Corvallis, OR, USA. [9]Soil and Environmental Sciences Department, University of Wisconsin, Madison, WI, USA. [10]USDA Forest Service Pacific Northwest Research Station, Portland, OR, USA. [11]U.S. Geological Survey, Washington Water Science Center, Tacoma, WA, USA. [12]Department of Civil and Environmental Engineering, The Pennsylvania State University, State College, PA, USA. [13]Department of Civil and Environmental Engineering, University of Louisville, Louisville, KY, USA. [14]Department of Environmental Sciences, University of Virginia, Charlottesville, VA, USA. [15]Biological Sciences, University of Alabama, Tuscaloosa, AL, USA. [16]Department of Civil and Environmental Engineering, Lafayette College, Easton, PA, USA. [17]U.S. Geological Survey, Wyoming-Montana Water Science Center, Helena, MT, USA. [18]Lane Ecological Advisory LLC, Pewaukee, WI, USA. [19]Biological and Ecological Engineering Department, Oregon State University, Corvallis, OR, USA.

Contents of this file

Text S1
Figures S1 to S15
Table S1 to S2

Introduction

This supporting information includes additional text and figures in support of the headwater types clustering and analysis. Stable links and DOIs for all data files and code can be found in the main document's availability statement.

We compiled watershed attributes (details in Table S2) and aggregated them at the HUC12 scale (Figure S1). We used these attributes to perform a clustering that identified groups of watersheds with similar profiles of watershed attributes (Text S1, Figure S2, Figure S3, Figure S4). We then compared the attributes of headwaters with downstream systems using a random forest classification model (Figure S5, Figure S6, Figure S7, Figure S11, Figure S12, Figure S13).

Figures S8-10 provide information on the distribution of all attributes used in the clustering for each headwater type.

Figure S14 shows how represented each headwater type is within long-term streamflow monitoring datasets (Sando et al., 2026).

Figure S15 shows a comparison of one NHD flow attribute (percent of stream kilometers classes as non-perennial) by headwater type.

Text S1.

Additional methods on clustering approach: We used k-means clustering (scikit-learn; Pedregosa et al., 2011) on normalized and transformed attributes to identify groups of headwaters with similar climatic, subsurface, and land-cover characteristics. K-means clustering is an unsupervised clustering algorithm that iteratively assigns observations to the nearest of *k* cluster centroids by minimizing within-cluster variance and recalculates centroids until convergence (MacQueen, 1967). Prior to clustering, the Yeo–Johnson transformation (Yeo & Johnson, 2000) was applied to correct skewness and standardize variables (zero mean, unit variance).

To identify the optimal number of clusters (k=1-15), we plotted the within-cluster sum of squares (WCSS) against increasing values of k to identify the "elbow" point where the rate of decrease in WCSS sharply diminishes, indicating that additional clusters yield diminishing returns in explained variance (Thorndike 1957, Figure S2). We corroborated this method using the silhouette score, which evaluates how well each observation fits within its assigned cluster relative to neighboring clusters (Figure S3). The optimal number of clusters is typically identified as the k that maximizes the mean silhouette score across all observations (Rousseeuw, 1987). Based on these assessments, we selected 7 clusters to balance parsimony and interpretability. Tests showed that 6–8 clusters produced similar spatial distributions (Figure S4), and using 8 clusters simply created smaller regional sub-groups. As in Wolock et al. (2004), our final classification combines data-driven clustering with expert interpretation to minimize within-cluster variance while ensuring broad applicability at the CONUS scale.

We identified 15 as an approximate upper limit on the number of clusters because it represented a mid-point between the number of hydrologic landscape regions identified in Wolock et al. 2004 (20 HLRs) and US EPA level 1 ecoregions (11 ecoregions in the continental US). We wanted to identify a relatively small number of clusters that would be interpretable, generalizable, and intuitive for end users. Therefore 15 seemed like a reasonable upper bound for the maximum number of clusters.

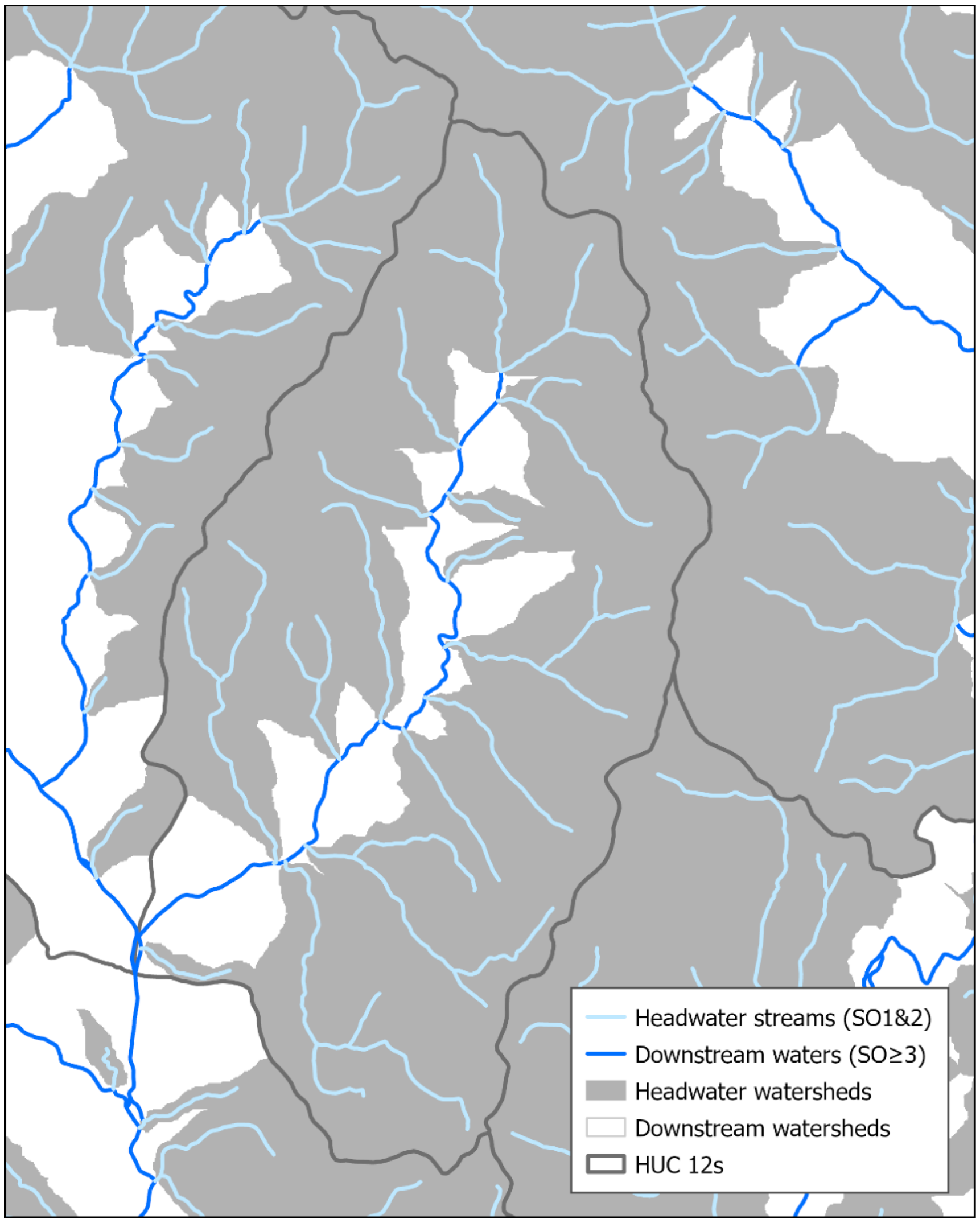

Figure S1. Example of one HUC12 watershed, Pocket Creek, TN (HUC 12 060200040203) split into headwaters (stream orders 1&2) and downstream (stream orders ≥3). Attributes are summarized for all headwaters and downstream within a given HUC12.

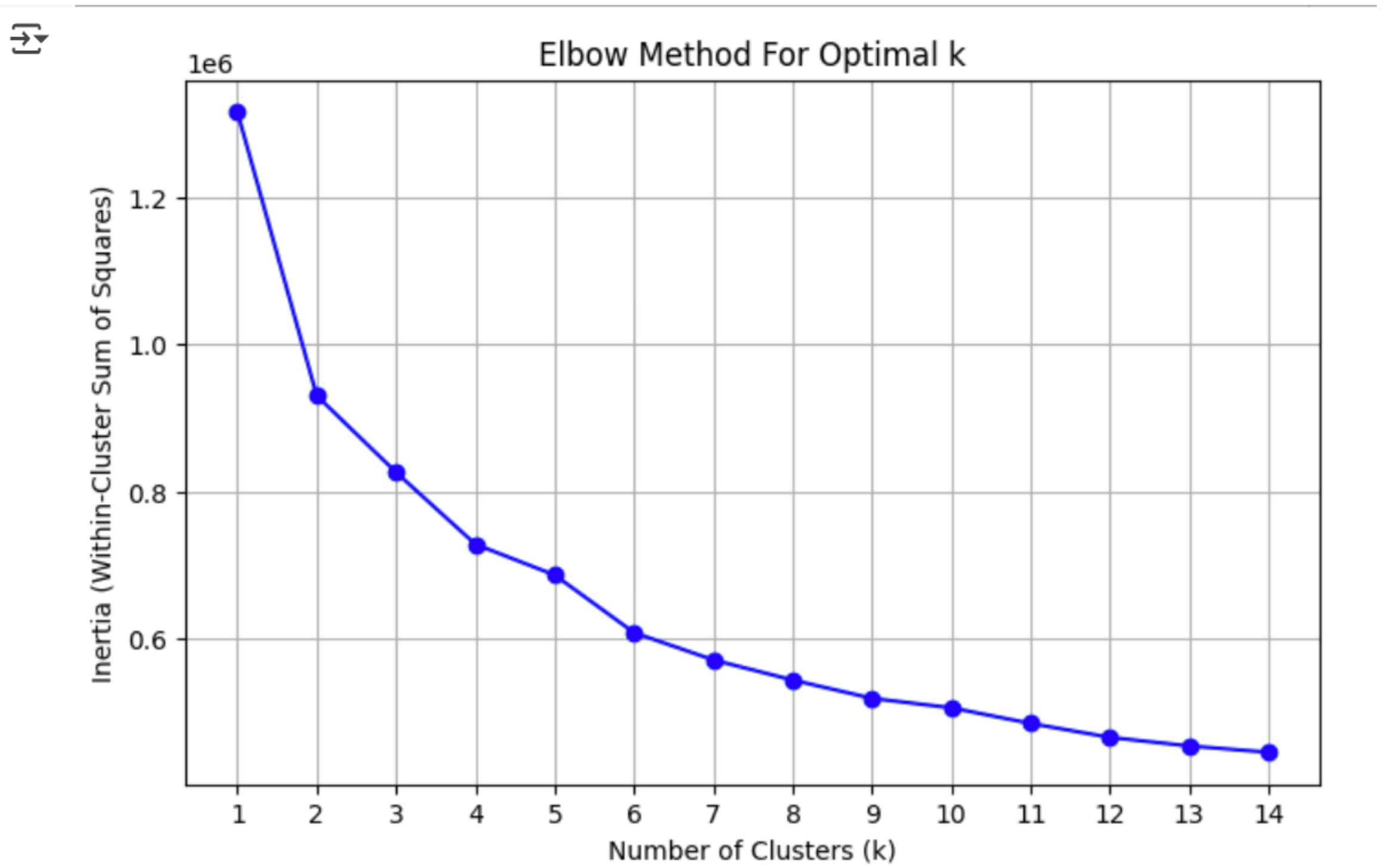

Figure S2. Elbow plot of the within-cluster sum of squares (WCSS) scores for varying numbers of clusters (k=1-14). WCSS is a measure of how tightly clustered data points are within each cluster. A lower WCSS indicates that the data points are more tightly grouped around their respective centroids.

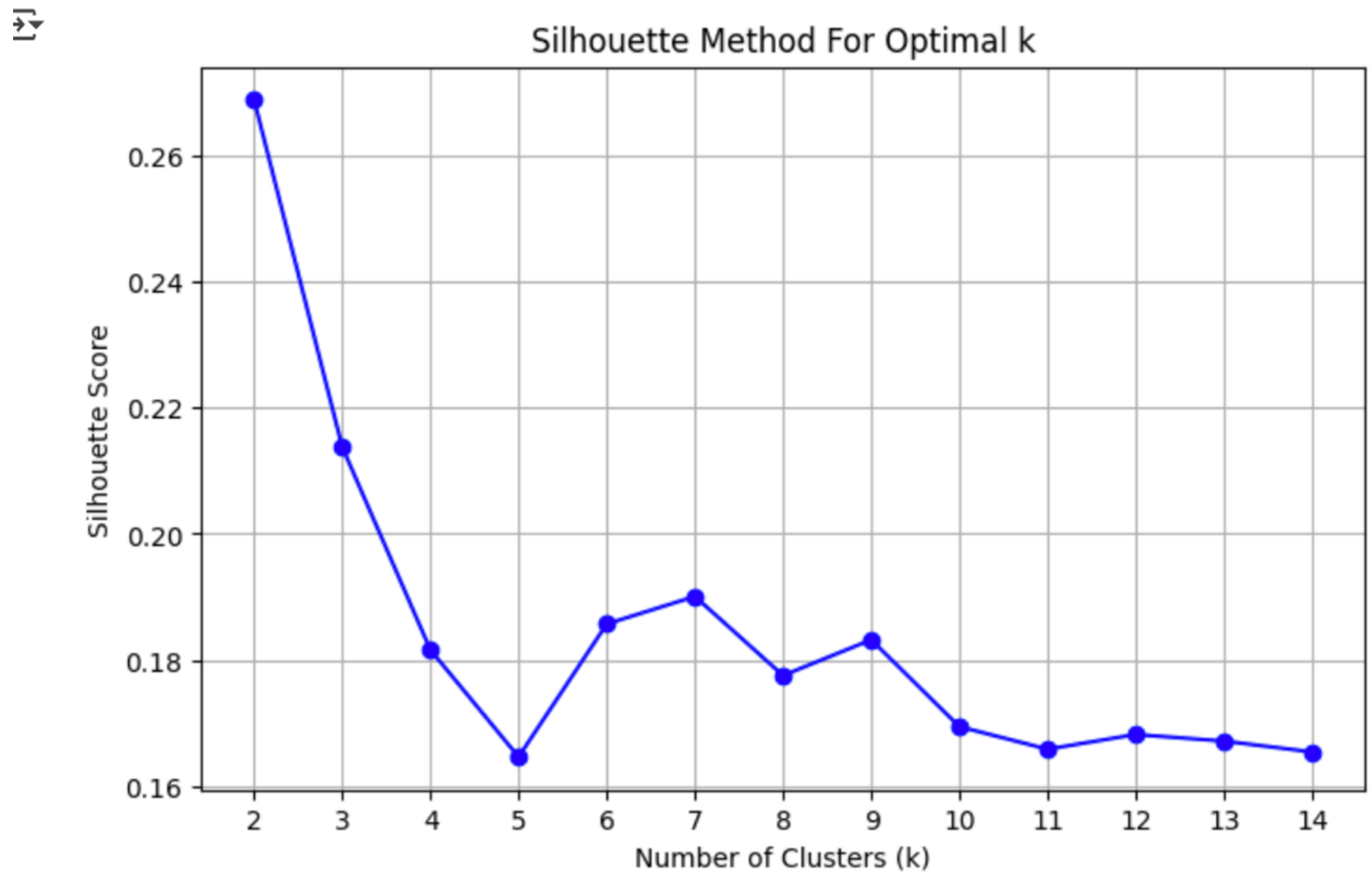

Figure S3. Plot of silhouette score for clusters k=1-14. The silhouette score measures how similar a data point is to its own cluster compared to other clusters. The optimal number of clusters will maximize the silhouette score.

a)

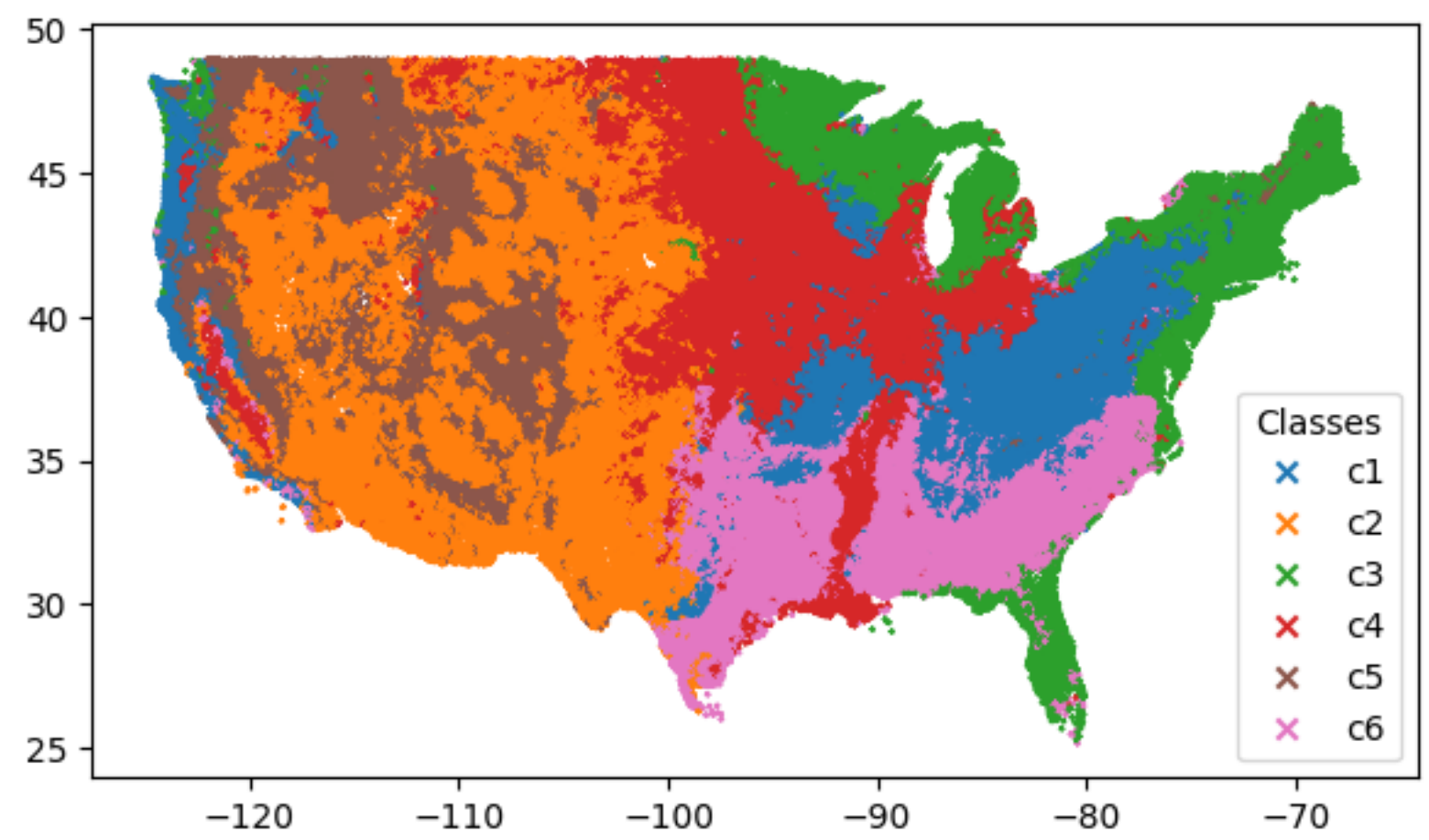

b)

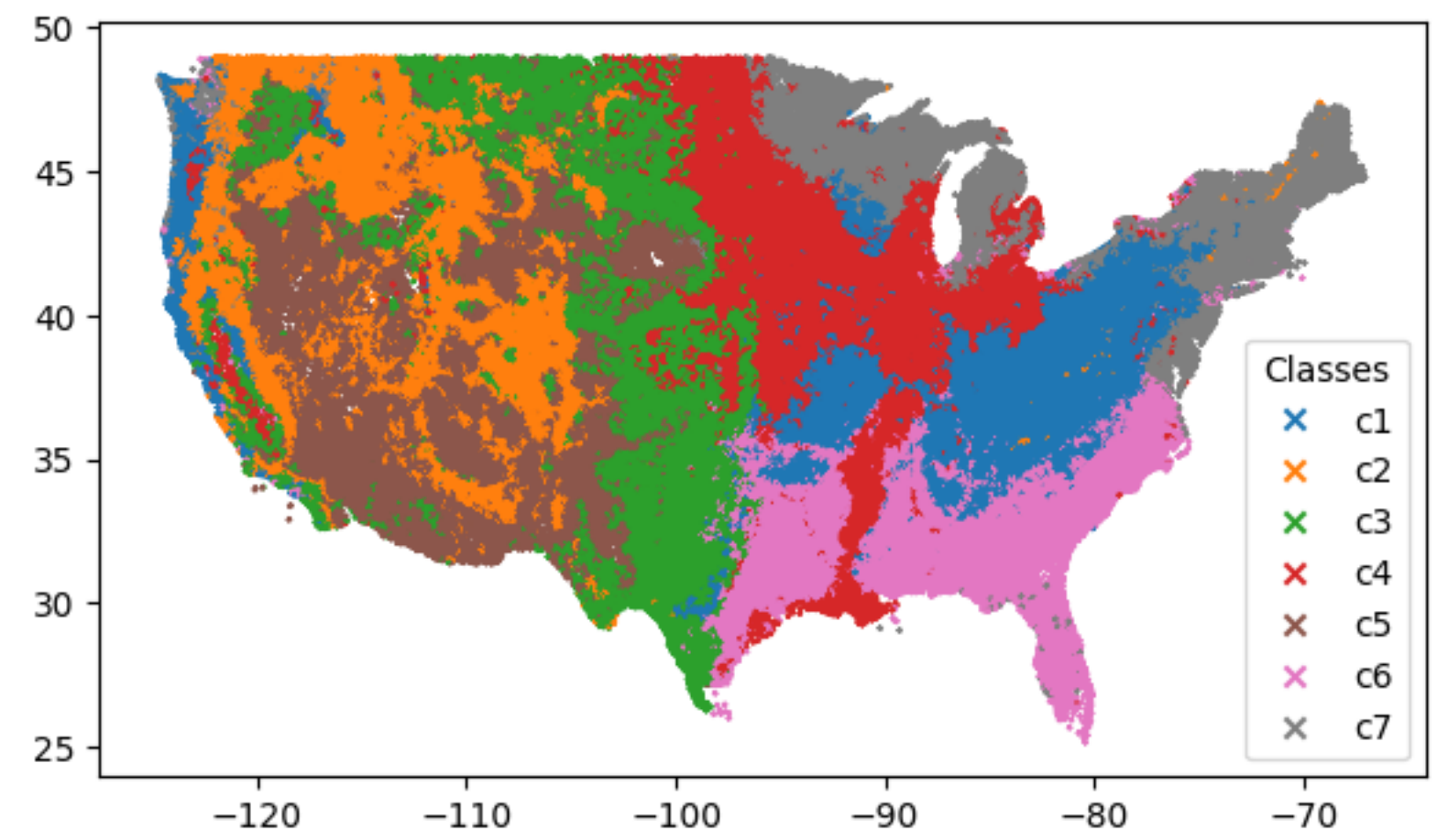

c)

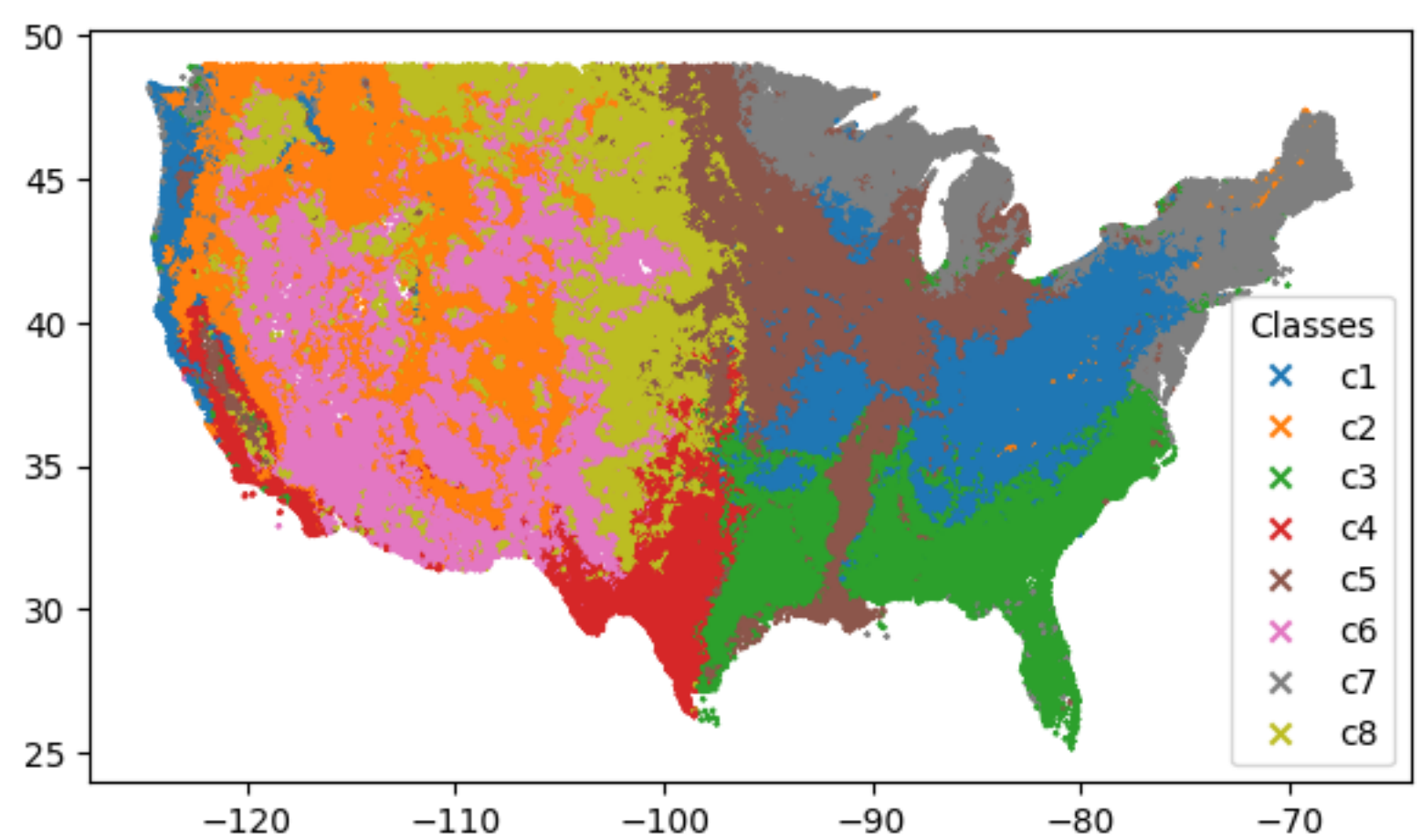

Figure S4. Comparison of k-means clustering results for k = 6, 7, and 8 clusters (panels, a, b, and c, respectively). The overall geographic distribution of clusters remained similar across this range of clusters but resulted in either lumping of very disparate areas (n=6 clusters; the northeastern US and Florida) and splitting of other more similar regions (n=8 clusters; separation of headwaters in Texas and the southeastern US).

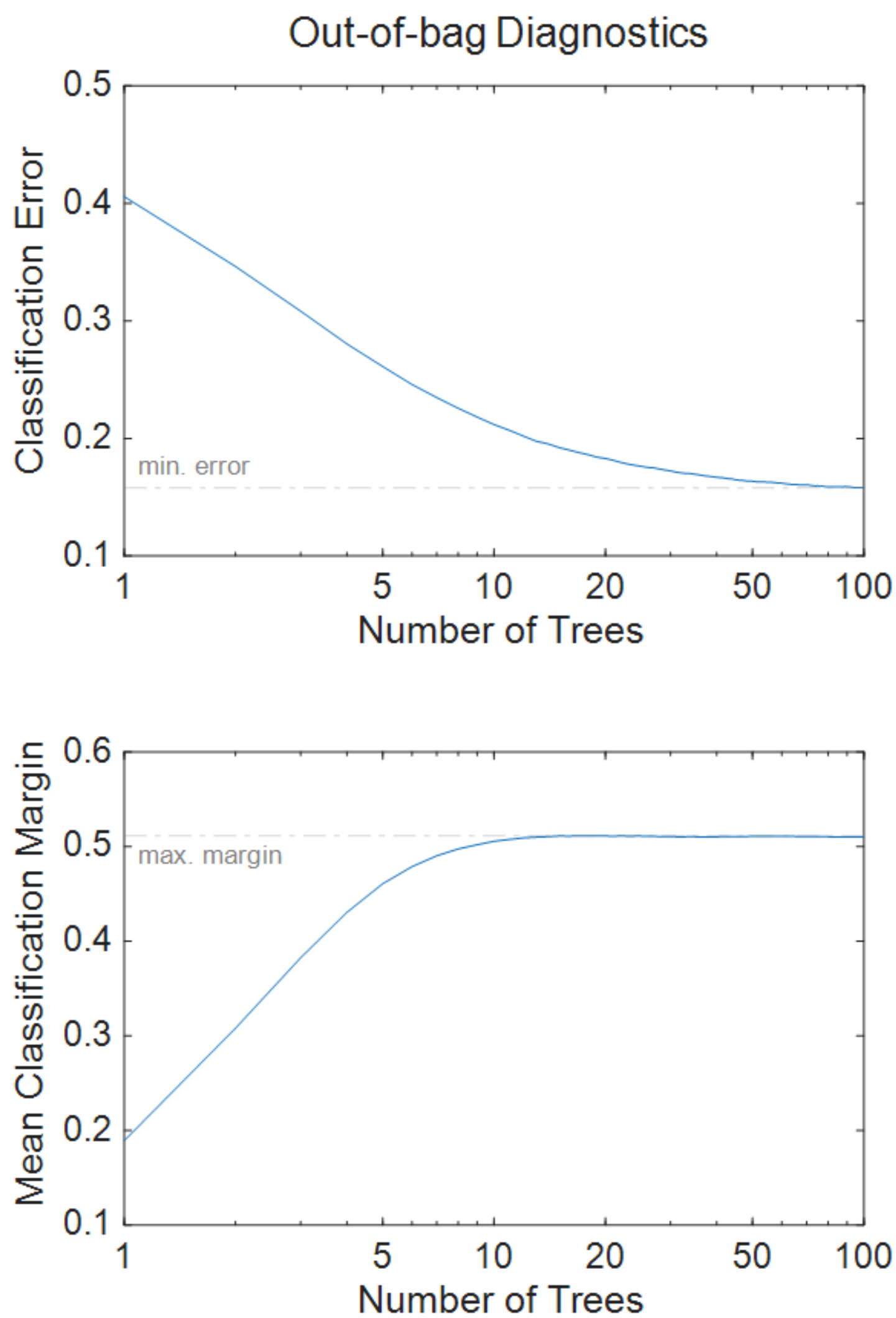

Figure S5. Out-of-bag learning curves for the classification ensemble model to distinguish headwater (stream orders 1&2) and downstream (stream orders ≥3) watersheds. Classification error shows the fraction of basins misclassified, which decreases as more trees are grown and stabilizes above 80 trees (top panel). Mean classification margin shows the average difference between the probability assigned to the true class and the competing class (bottom panel). Most observations receive confident ensemble votes after 20 trees have been grown.

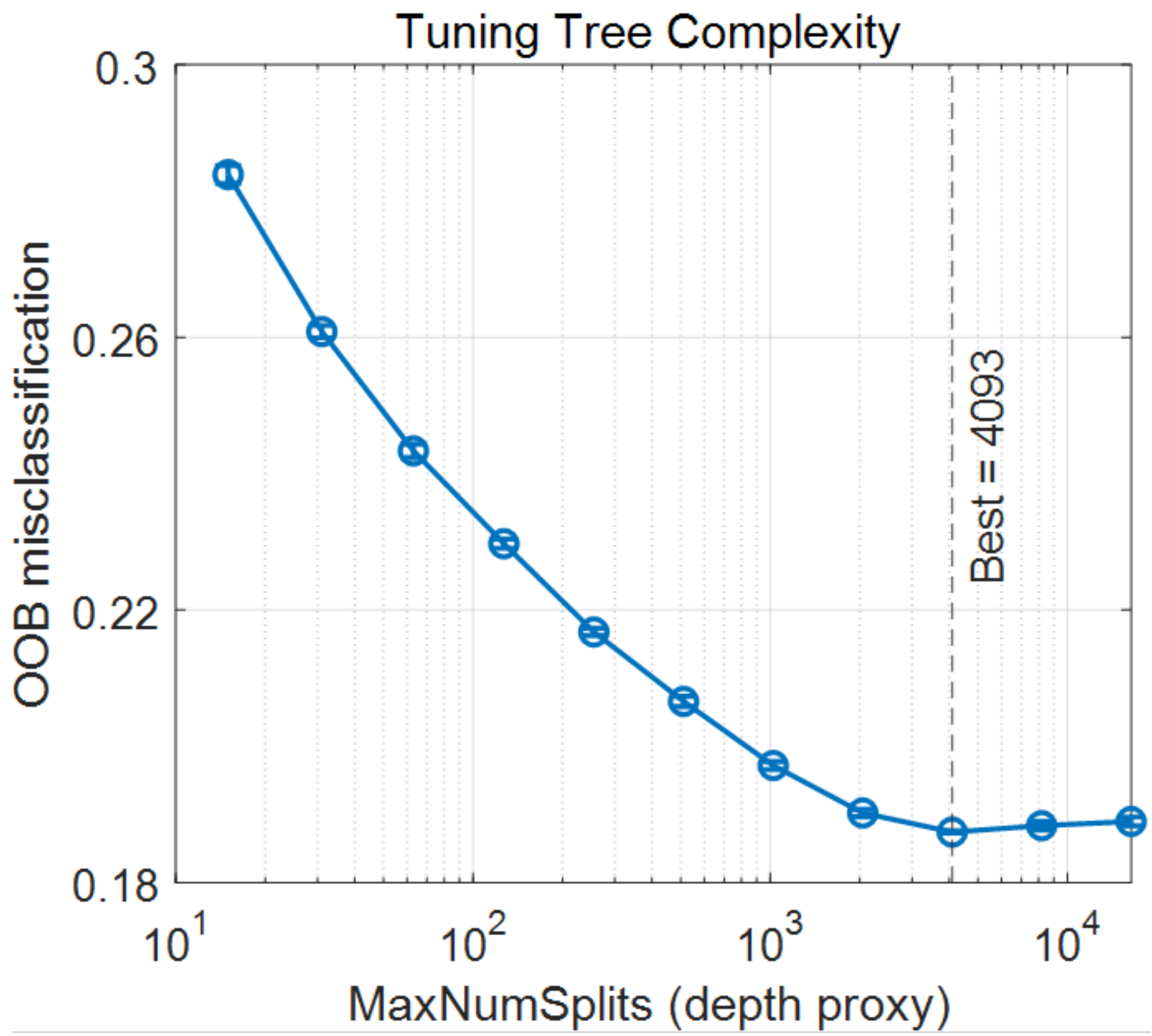

Figure S6. Classification model tuning through evaluation of multiple candidate MaxNumSplits (the maximum number of decision splits or branching points), which are a proxy for tree depth. Out-of-bag (OOB) classification error shows the fraction of basins misclassified. The minimum for OOB misclassification is identified by a "Best" line.

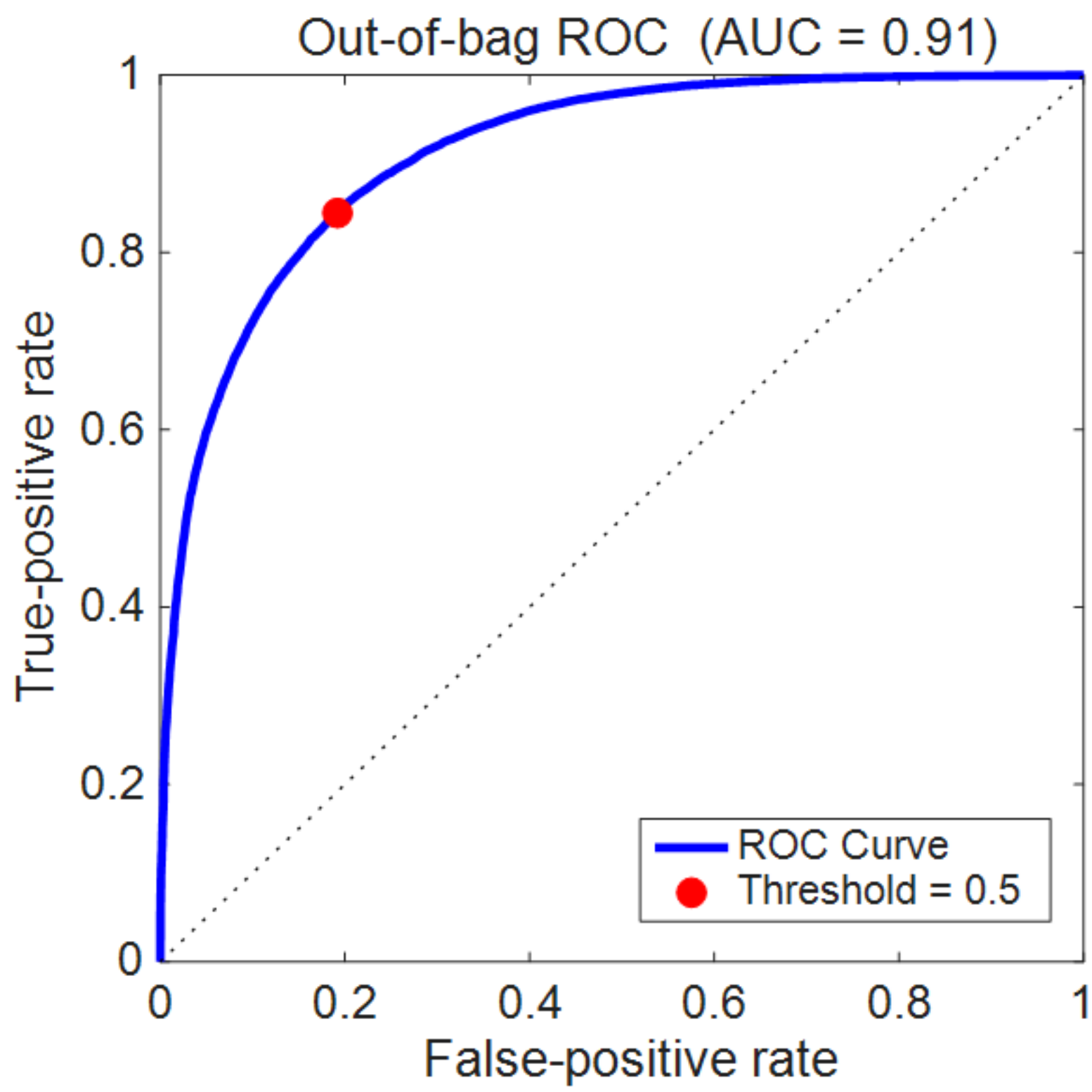

Figure S7. Out-of-bag receiver-operating-characteristic (ROC) curve for the ensemble model that classifies watersheds as headwater (stream orders 1&2) or downstream (stream orders ≥3). The blue line shows the trade-off between the true-positive (TPR) and false-positive (FPR) rates. The model's discrimination ability is summarized by the area under the curve (AUC = 0.91), suggesting strong performance (1.0 = perfect discrimination, 0.5 = random guessing). The red marker represents the performance (TPR = 0.84, FPR = 0.19) of the entire ensemble model at the default 0.5 decision threshold. The one-to-one diagonal line indicates the performance of a no-skill classifier.

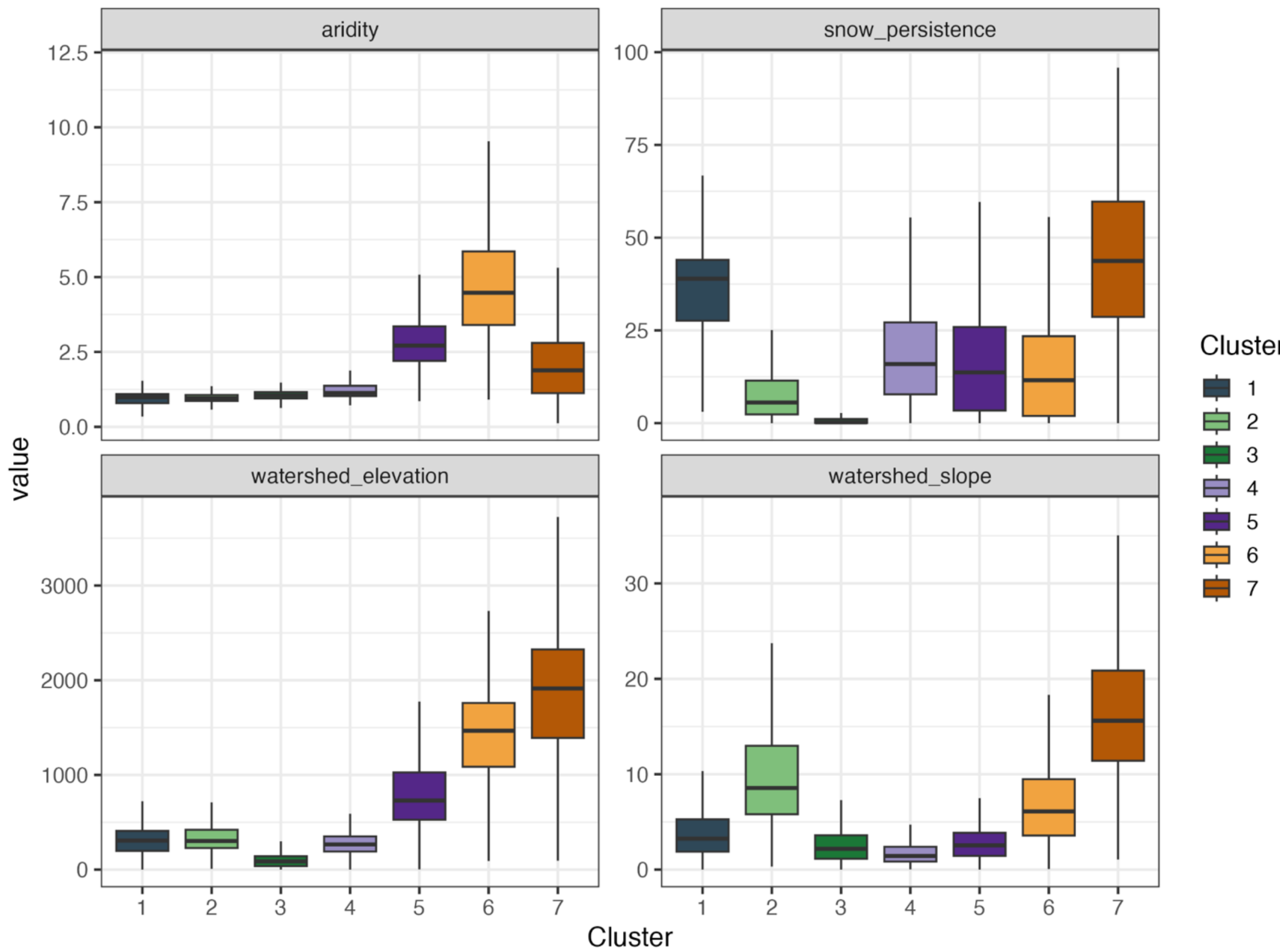

Figure S8. Boxplots of climate and topographic attributes by cluster. Boxplot line is the median and box extent is 75th and 25th percentile extent, respectively with whiskers representing the 90th and 10th percentiles, respectively.

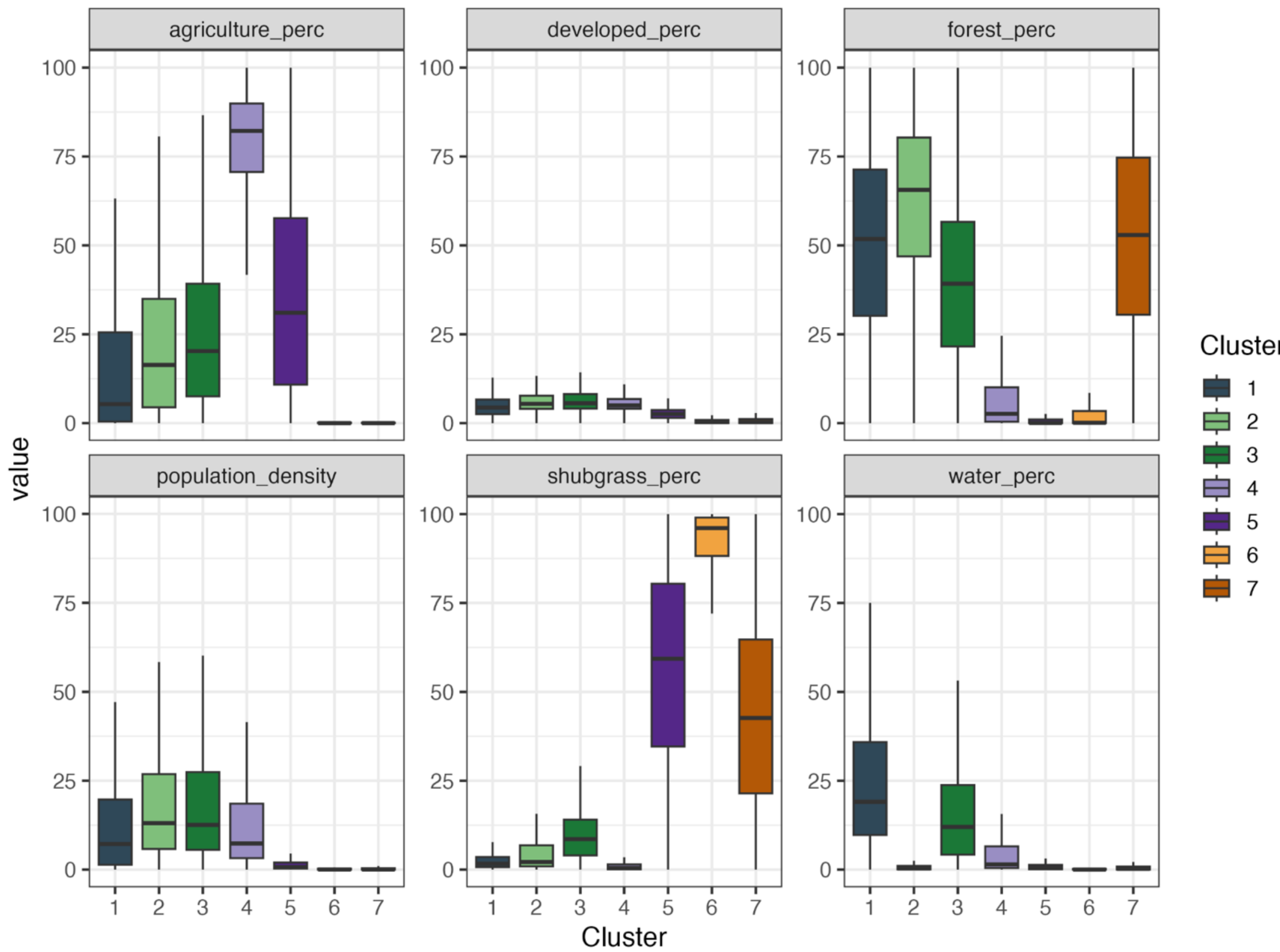

Figure S9. Boxplots of land cover and human impact attributes by cluster. Boxplot line is the median and box extent is 75th and 25th percentile extent, respectively with whiskers representing the 90th and 10th percentiles, respectively.

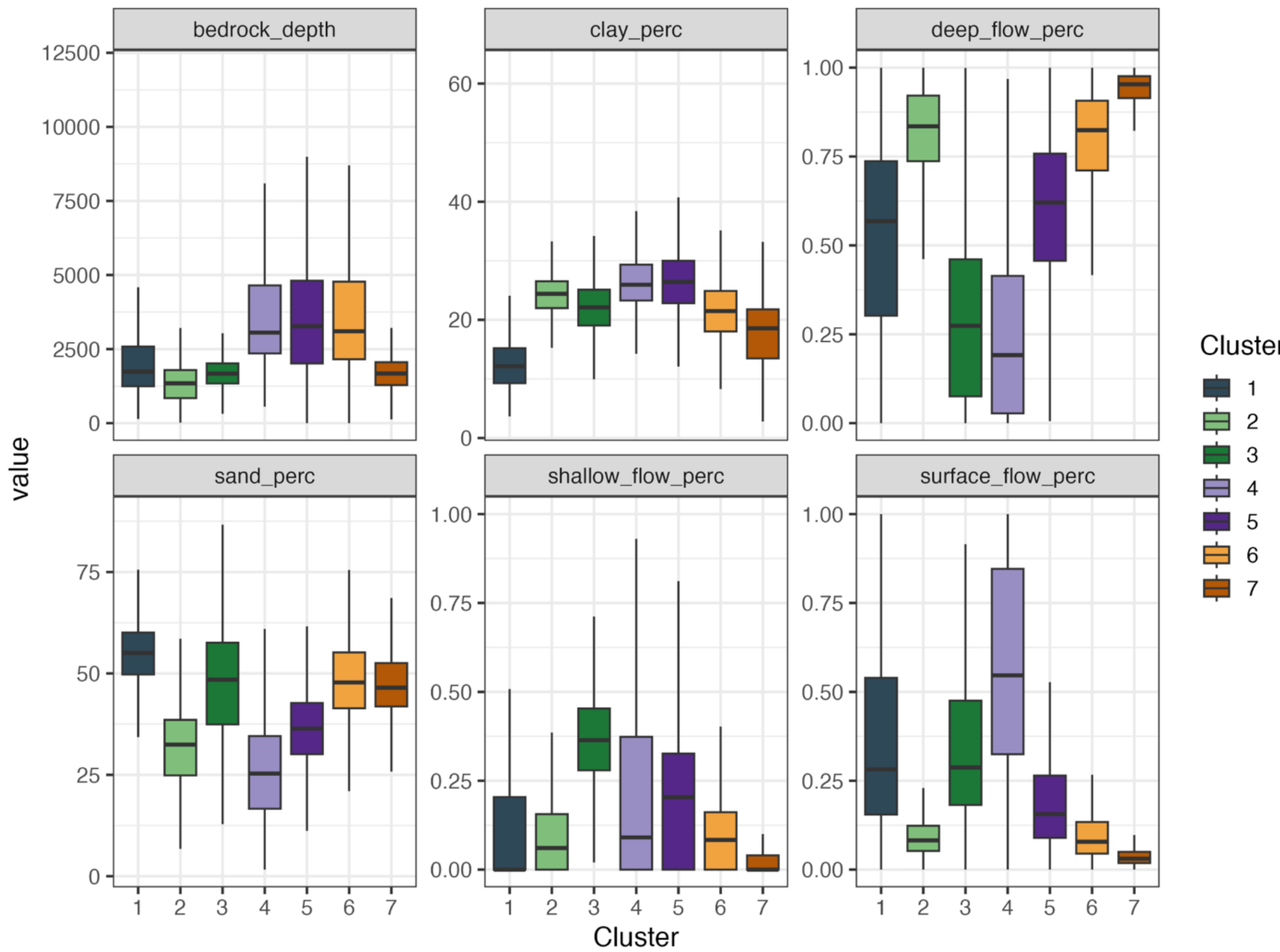

Figure S10. Boxplots of soil and bedrock variables and flowpath depth (proxied by HAND class fractions) by cluster. Boxplot line is the median and box extent is 75th and 25th percentile extent, respectively with whiskers representing the 90th and 10th percentiles, respectively.

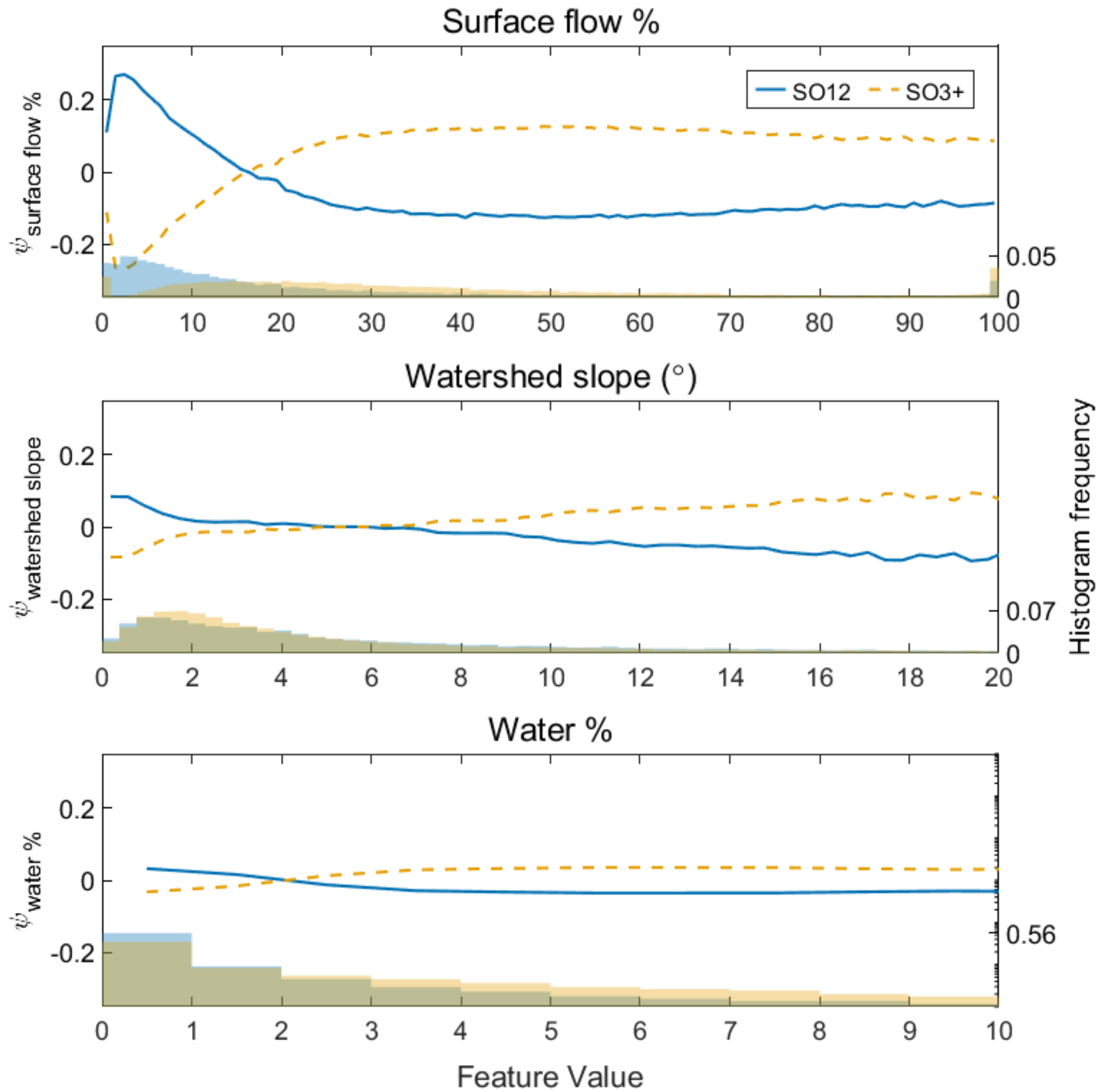

Figure S11. Shapley dependence plots for three influential watershed attributes, indicating the probability of a site being classified as either a headwater (stream orders 1&2; SO12) or downstream (stream orders ≥3; SO3+). The lines (left y-axis) show the Shapley values (ψ) (Lundberg & Lee, 2017; Husic et al., 2025), which represent the marginal contribution of a feature to the probability score for classification of SO12 or SO3+; positive values indicate a positive influence on the probability for that class. The histograms (right y-axis) depict the probability distribution of each feature for headwater and downstream sites. Note: the histogram scale for "Water %" is logarithmic to visualize distribution of values that are obscured in a linear scale.

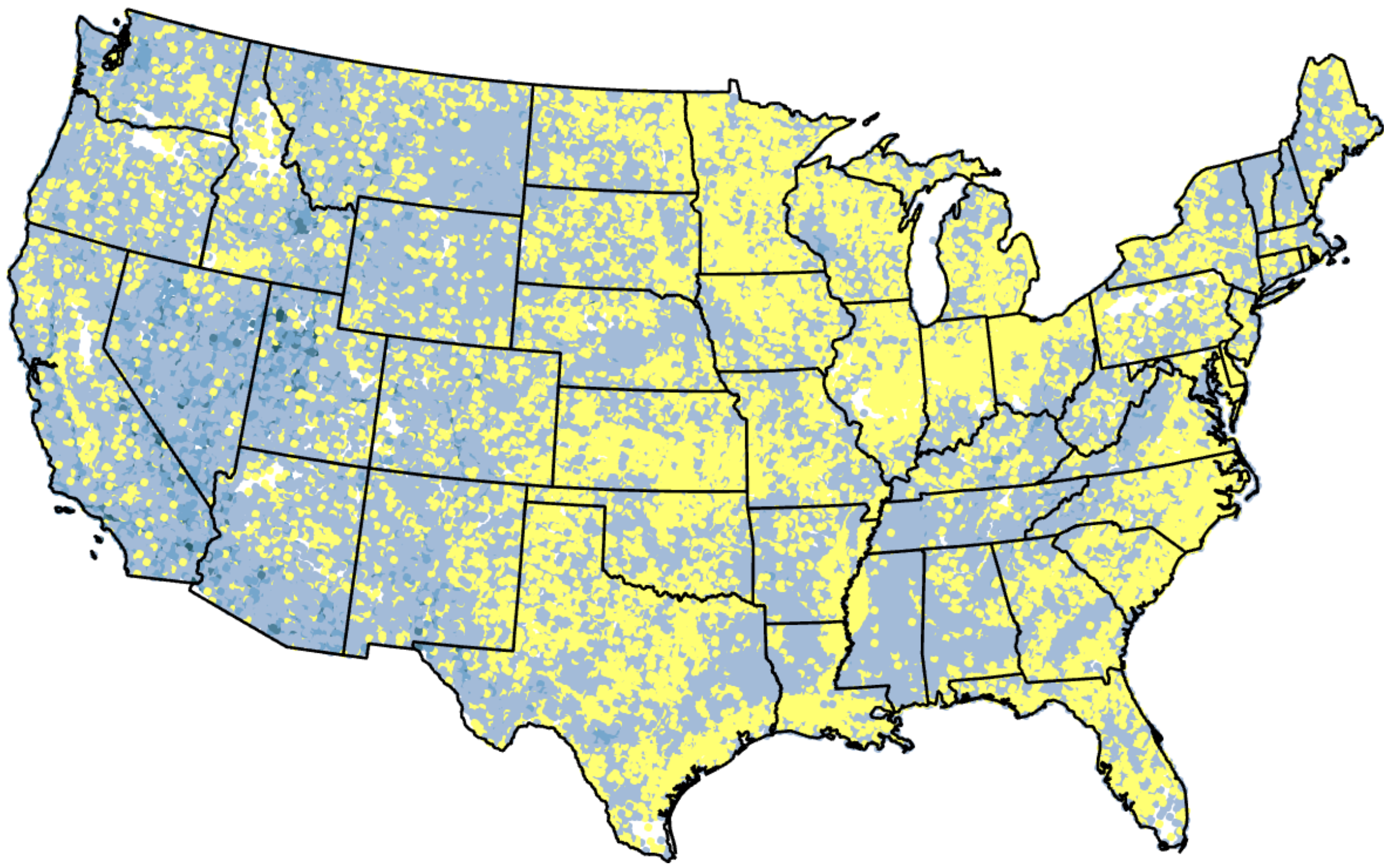

Figure S12. Map of HUC12s included in analysis colored by the difference between mean watershed slope in headwater (stream orders 1&2) and downstream (stream orders ≥3) portions of the HUC12. Grey colors indicate HUC12s where mean watershed slope is greater in the headwater system, whereas yellow colors indicate HUC12s where mean watershed slope is greater in the downstream system.

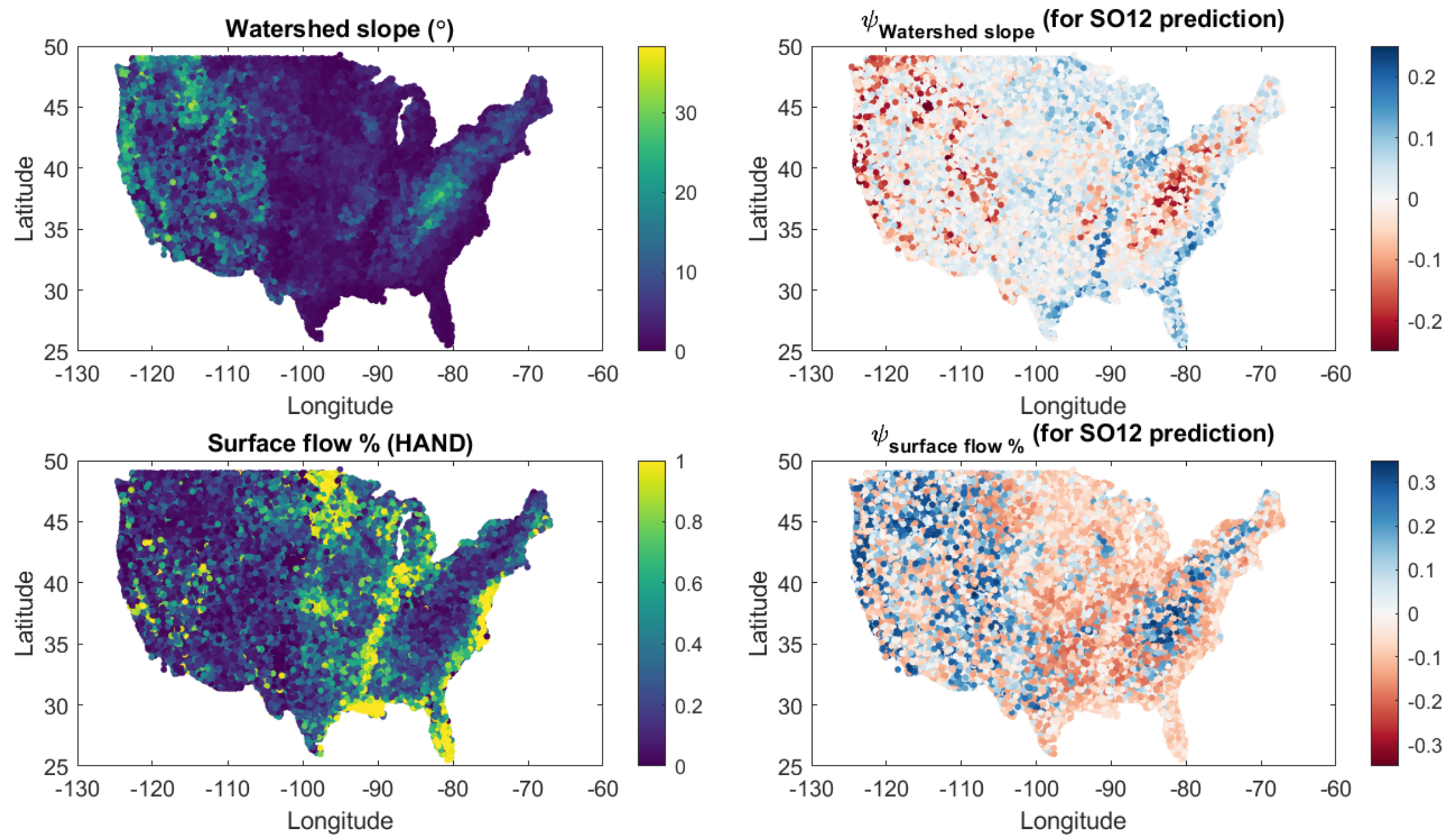

Figure S13. Top row: Watershed slope feature map (left panel) and watershed slope feature Shapley values for SO12 prediction (right panel). Bottom row: Surface flow % HAND feature map (left panel) and Surface flow % Shapley values for SO12 prediction (right panel).

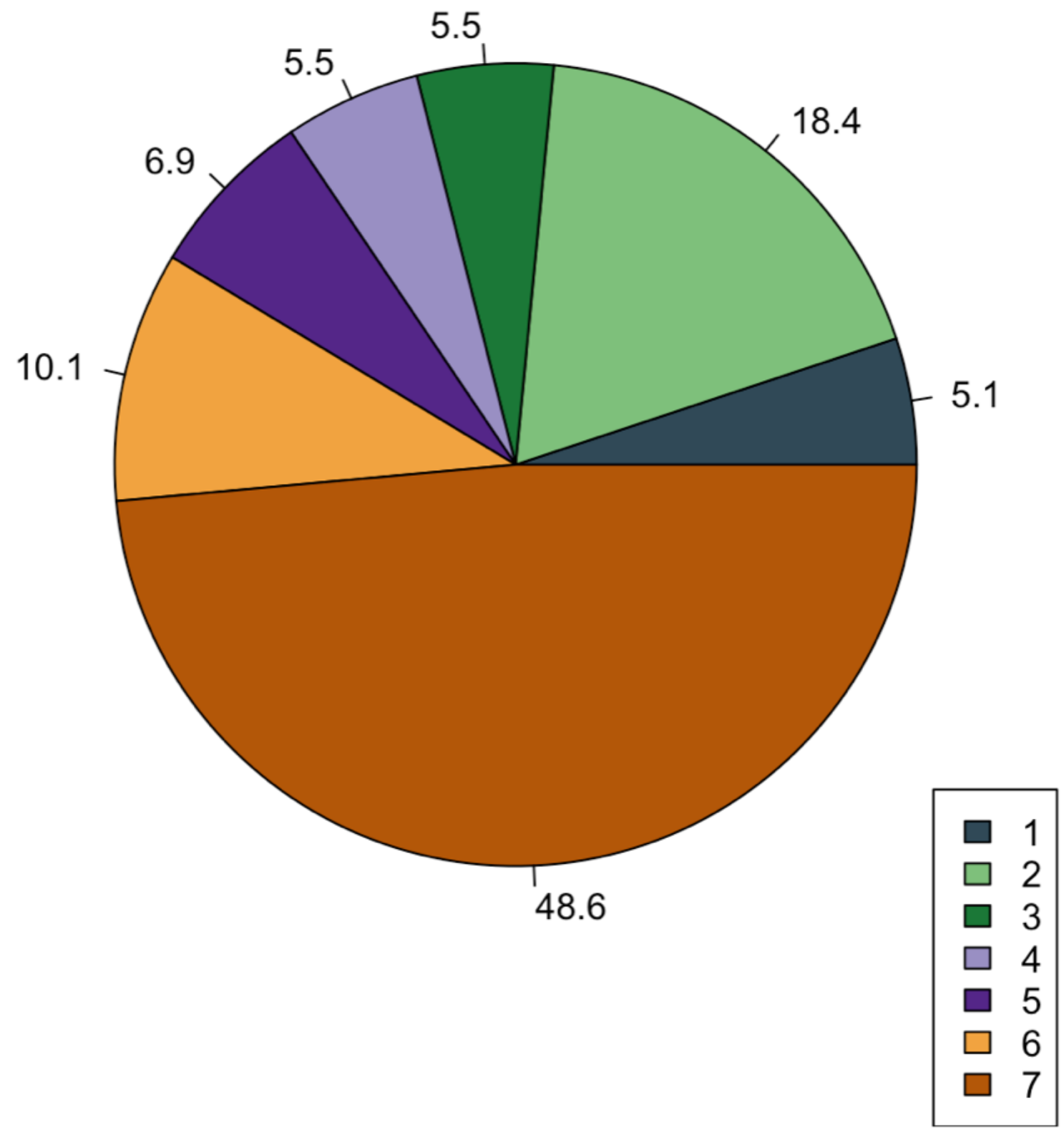

Figure S14. Percent of stream monitoring locations identified in Sando et al. (2026) in each headwater type. We cross referenced each stream monitoring location (n=117,675) with the HUC12 watershed boundaries/headwater types determined in this analysis to identify which types had the greatest share of long-term monitoring data. The headwater type is indicated by color and labeled with the percentage of monitoring locations in that type.

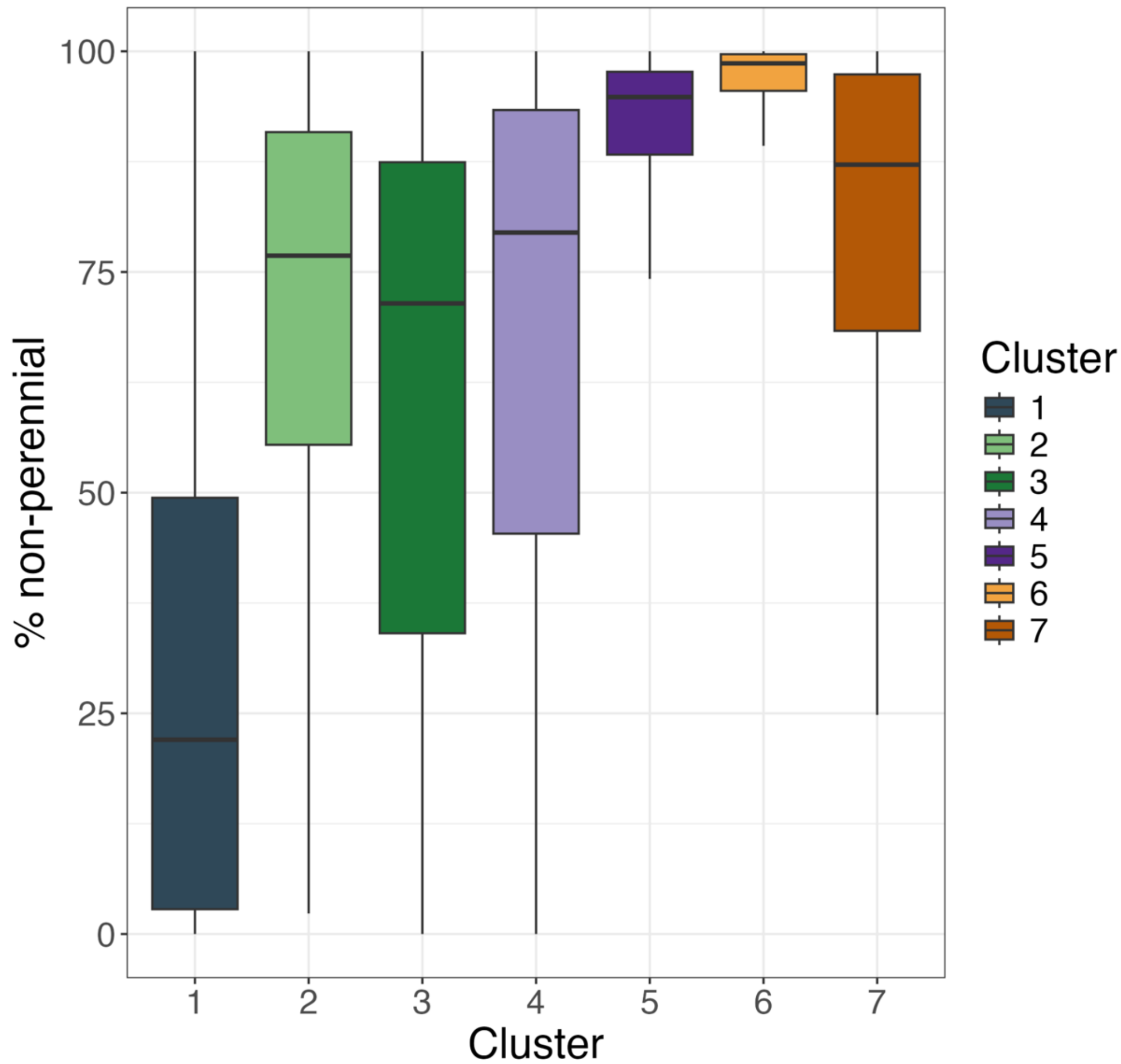

Figure S15. Percent of stream kilometers in headwaters (stream orders 1&2) that were flagged as non-perennial (sum of intermittent and ephemeral) in the NHDPlus-HR F-code dataset.

Table S2. Watershed attributes used in clustering and random forest analysis.

Climate	Description	Method of calculation	Source	Spatial Res.	Citation	URL
Snow Persistence	Index describing the fraction of time that snow is present on the ground between January 1 and July 3.	Long-term mean average; spatially averaged over entire catchment area	USGS	500m	Hammond et al. 2020	https://doi.org/10.5066/P9U7U5FP
Aridity	The ratio of mean annual precipitation (P) to mean annual potential evapotranspiration (PET).	Long-term mean average; spatially averaged over entire catchment area	gridMET	4000m	Abatzoglou 2011	https://doi.org/10.1002/joc.3413
Topography, Soils, Geology						
Watershed slope	Mean watershed slope derived from NHDHR DEM using the terra package in R	Spatially averaged over entire catchment	USGS	10m	USGS 2019	https://apps.nationalmap.gov/downloader/#/
Watershed elevation	Mean watershed elevation obtained from the NHD-HR DEM	Spatially averaged over entire catchment	USGS	10m	USGS 2019	https://apps.nationalmap.gov/downloader/#/
% Sand	Mean % sand fraction at X depths	Spatially averaged over entire catchment	SoilGrids	250m	Batjes et al. 2020	https://soilgrids.org/
% Clay	Mean % clay fraction at X depths	Spatially averaged over entire catchment	SoilGrids	250m	Batjes et al. 2020	https://soilgrids.org/
Bedrock Depth	Mean absolute depth to bedrock (in cm) predicted using the global compilation of soil ground observations.	Spatially averaged over entire catchment	SoilGrids	250m	Batjes et al. 2020	https://soilgrids.org/
Land Cover and Human Alteration						
% water	% of total watershed area comprised of this combined land cover class; sum of open water class and wetland class (woody and herbaceous)	The % of total pixels in the catchment assigned as this land cover class	NLCD 2021	30m	USGS 2024	https://www.mrlc.gov/data
% developed	% of total watershed area comprised of this combined land cover class; sum of all developed sub-classes (open, low intensity, medium intensity, and high intensity)	The % of total pixels in the catchment assigned as this land cover class	NLCD 2021	30m	USGS 2024	https://www.mrlc.gov/data
% forest	% of total watershed area comprised of this combined land cover class; sum of mixed forest, evergreen forest, deciduous forest classes	The % of total pixels in the catchment assigned as this land cover class	NLCD 2021	30m	USGS 2024	https://www.mrlc.gov/data
% shrub/grass	% of total watershed area comprised of this combined land cover class; sum of all herbaceous classes (grassland/herbaceous, sedge/herbaceous) and shrubland classes (dwarf scrub and shrub/scrub)	The % of total pixels in the catchment assigned as this land cover class	NLCD 2021	30m	USGS 2024	https://www.mrlc.gov/data
% agriculture	% of total watershed area comprised of this combined land cover class; sum of pasture/hay class and cultivated crop class	The % of total pixels in the catchment assigned as this land cover class	NLCD 2021	30m	USGS 2024	https://www.mrlc.gov/data
Population density	Population sum (derived from disaggregated census data, Huang et al. 2020) normalized by watershed area	Sum of all pixels within entire catchment divided by catchment area	Huang et al. 2020	100m	Huang et al. 2020	https://doi.org/10.1080/20964471.2020.1776200.
Hydrology						
% surface HAND	HAND describe the relative soil gravitational potentials or the local drainage potentials; a pixel was classified as surface if the HAND value was 0-5m	The % of total pixels in the catchment assigned as surface HAND pixels	Liu 2023	10m	Liu et al. 2023	https://doi.org/10.4211/hs.73aaa3efcda2465ba6227f535400f36b
% shallow HAND	HAND describe the relative soil gravitational potentials or the local drainage potentials; a pixel was classified as surface if the HAND value was 5-15m	The % of total pixels in the catchment assigned as shallow HAND pixels	Liu 2023	10m	Liu et al. 2023	https://doi.org/10.4211/hs.73aaa3efcda2465ba6227f535400f36b
% deep HAND	HAND describe the relative soil gravitational potentials or the local drainage potentials; a pixel was classified as surface if the HAND value was greater than 15m	The % of total pixels in the catchment assigned as deep HAND pixels	Liu 2023	10m	Liu et al. 2023	https://doi.org/10.4211/hs.73aaa3efcda2465ba6227f535400f36b

Table S1. Assessment of forest cover for watersheds in the McMillan et al. 2023 publication. We filtered the entire McMillan et al. dataset (n=63) to identify 1) watersheds in the continental United States, that were 2) over 0.2 km^2, and 2) where we could successfully delineate a watershed boundary (n=51). We then used NLCD to estimate the % forest cover (defined as XXX classes) and determine whether forested landcover was the majority land cover class. This is reflected in the "Majority forested" column. We also used an approximate 20 km^2 threshold to indicate watersheds in this dataset that were also likely headwater watersheds ("HW" column).

This analysis shows that 31 out of 51 watersheds were majority forested (61%). Within the forested watershed subset, median % forest cover was 98%, and mean % forest cover was 86%. The % of watersheds with majority forested landcover increases slightly when looking at watersheds under 20 km^2 from 61% to 66%. Many of these forested watersheds appear to be located at CZOs, LTERs, or other experimental or research watersheds (28 out of 31; 90%), which further suggests minimal human impact.

Watershed Name	Area (km^2)	% Tree Cover	Majority Forested?	HW?
Upper Fourmile Branch, South Carolina	0.35	91.1	yes	yes
Archer Creek watershed, New York	1.32	100.0	yes	yes
Augusta Creek, Michigan	89.60	50.3	yes	no
Buck Creek, New York	3.04	100.0	yes	yes
Bull&Providence catchments, Kings River, California	2.44	99.1	yes	yes
Casper Creek, California	19.86	99.7	yes	yes
Catskill Mountains, New York	28.01	92.3	yes	no
Coweeta, North Carolina	16.53	99.5	yes	yes
Duke Forest Research Watershed, North Carolina	0.10	100.0	yes	yes
Eel River CZO - Angelo	16.69	99.3	yes	yes
Falling Rock, Robinson Forest, Kentucky	1.05	100.0	yes	yes
H.J. Andrews, Oregon	61.14	73.0	yes	yes
Hubbard Brook, New Hampshire	31.74	97.6	yes	yes
Irondequoit Creek basin, New York	102.64	40.3	yes	no
Little River Experimental Watershed, Georgia	342.09	45.1	yes	no
Marshall Gulch, Santa Catalina Mountains CZO	1.56	35.4	yes	yes
Neversink River watershed, Catskills, New York	243.84	93.8	yes	no
North Fork Caspar Creek watershed, California	5.33	100.0	yes	yes
Oak Ridge Reservation, Tennessee	1380.61	62.0	yes	no
Panola Mountain Research Watershed, Georgia	0.44	95.5	yes	yes
Santee, South Carolina	1.85	100.0	yes	yes
Shelter Creek, New York	1.86	100.0	yes	yes
Sleepers River, Vermont	111.26	84.8	yes	yes
Susquehanna Shale Hills CZO - Garner Run, Pennsylvania	1.92	89.2	yes	yes
W2; H.J. Andrews, Oregon	0.58	96.0	yes	yes
Walker branch watershed, Tennessee	1.21	100.0	yes	yes
WS1, Hubbard Brook Experimental Forest, New Hampshire	0.14	100.0	yes	yes
WS02, Coweeta, North Carolina	0.14	96.5	yes	yes
WS10, H.J. Andrews, Oregon	0.10	100.0	yes	yes
WS3, Hubbard Brook Experimental Forest, New Hampshire	0.40	98.9	yes	yes
WS6; Hubbard Brook, New Hampshire	0.15	99.9	yes	yes
Blacklands Experimental Watershed, USDA-ARS, Texas	0.60	0.0	no	yes

Watershed Name	Area (km^2)	% Tree Cover	Majority Forested?	HW?
Eel River CZO - Sagehorn	9.40	36.9	no	yes
GL4 catchment, Green Lakes Valley, Colorado	0.36	8.6	no	yes
Goodwater Creek, Missouri	72.16	7.9	no	no
Konza Prairie LTER, Kansas	13.23	3.2	no	yes
Loch Vale watershed, Colorado	6.92	9.1	no	yes
Logan River watershed, Utah	1363.06	39.4	no	no
Martinelli catchment, Green Lakes Valley, Colorado	0.47	32.5	no	yes
Meadowbrook Creek, New York	11.29	3.7	no	yes
Río Puerco, New Mexico	16639.08	6.3	no	no
Saddle Catchment, Niwot Ridge LTER, Colorado	0.31	20.2	no	yes
San Diego River watershed, California, United States	1115.21	11.6	no	no
San Dimas, California	1.01	0.1	no	yes
Upper Dry Creek, near Boise, Idaho	0.16	7.0	no	yes
Upper Sheep Creek, Reynolds Creek, Idaho	0.41	0.0	no	yes
Valles Caldera, Jemez, New Mexico	448.40	38.2	no	no
Walnut Gulch Experimental Watershed, Arizona	150.28	0.0	no	no
Watershed N04D, Konza Prairie LTER, Kansas	1.59	0.0	no	yes
WE-38, Mahantango Creek Experimental Catchment, Pennsyl	7.20	38.2	no	yes
Yucca Mountain, Nevada	41.96	0.0	no	no
